# Supplementary material for: Single-Cell DNA Methylome Sequencing and Bioinformatic Inference of Epigenomic Cell-State Dynamics
Source: Cell Rep. 2015 Feb 26;10(8):1386–97. doi: 10.1016/j.celrep.2015.02.001 (PMC4542311; doi:10.1016/j.celrep.2015.02.001)
Supplement: Document S1. Supplemental Experimental Procedures, Figures S1–S6, and Table S2 [file mmc1.pdf]

Cell Reports

Supplemental Information

Single-Cell DNA Methylome Sequencing and Bioinformatic Inference of Epigenomic Cell-State Dynamics

Matthias Farlik, Nathan C. Sheffield, Angelo Nuzzo, Paul Datlinger, Andreas Schönegger, Johanna Klughammer, and Christoph Bock

Supplemental Figures and Tables

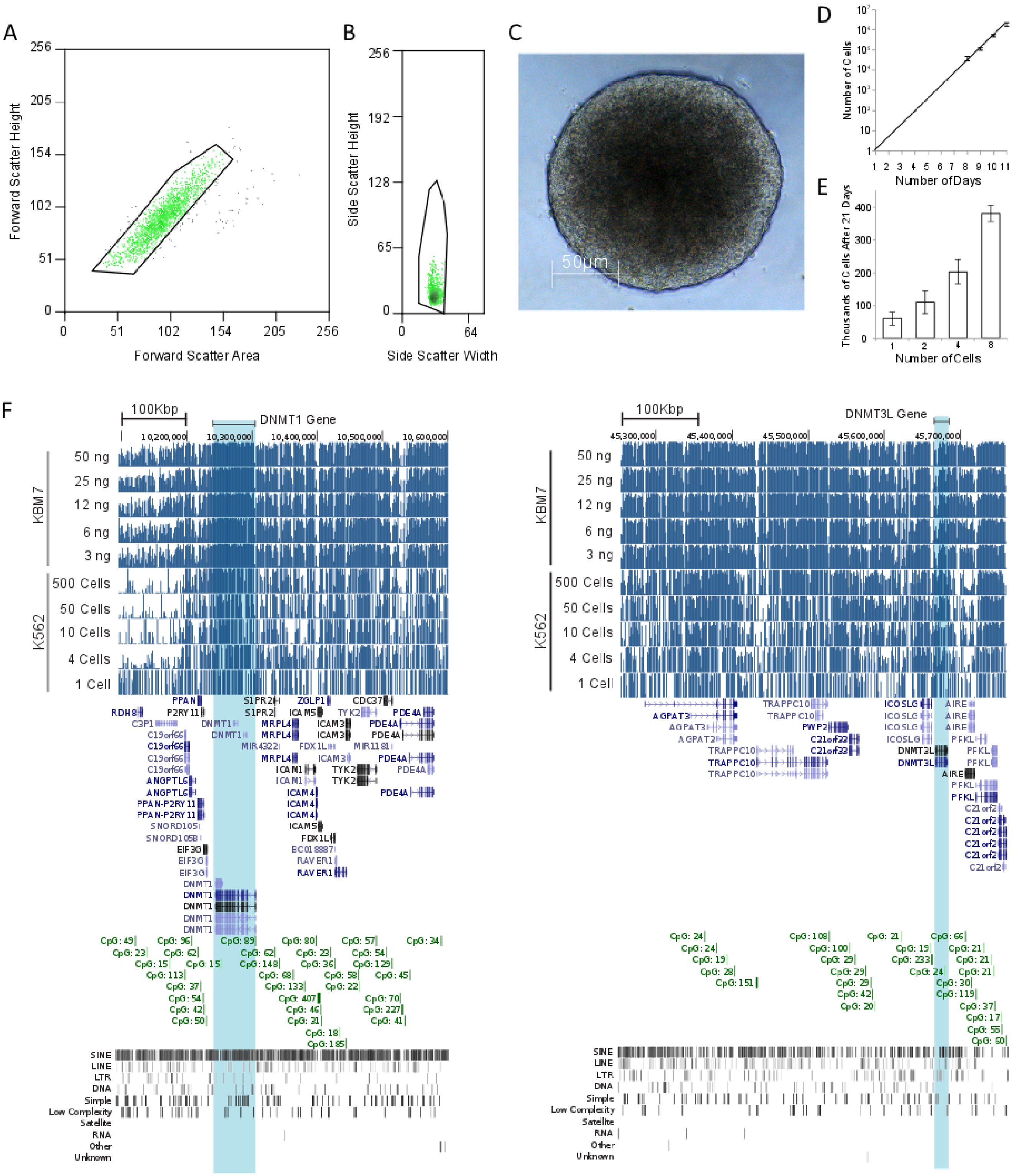

Figure S1. Validation of single-cell sorting, Related to Figure 1

(**A**) Single K562 cells were distinguished from cell doublets (which are visible as a small cloud on the right) based on forward scatter height and forward scatter area. (**B**) Gated single cells were plotted again according to side scatter height and side scatter width (for validation purposes). (**C**) Picture of a representative, single-cell derived ES cell colony. (**D**) The growth of 32D cells was monitored over a period of 11 days. Error bars denote the standard deviation of 10 individual colonies. (**E**) K562 cell growth was monitored for 21 days after initial seeding of 1, 2, 4, or 8 cells. Error bars indicate standard deviation of 10 individual colonies. These results confirm the robustness of the single-cell sorting. (**F**) DNA methylation profiles of two 500-kilobase genomic regions surrounding the DNMT1 gene (left panel) and the DNMT3L gene (right panel).

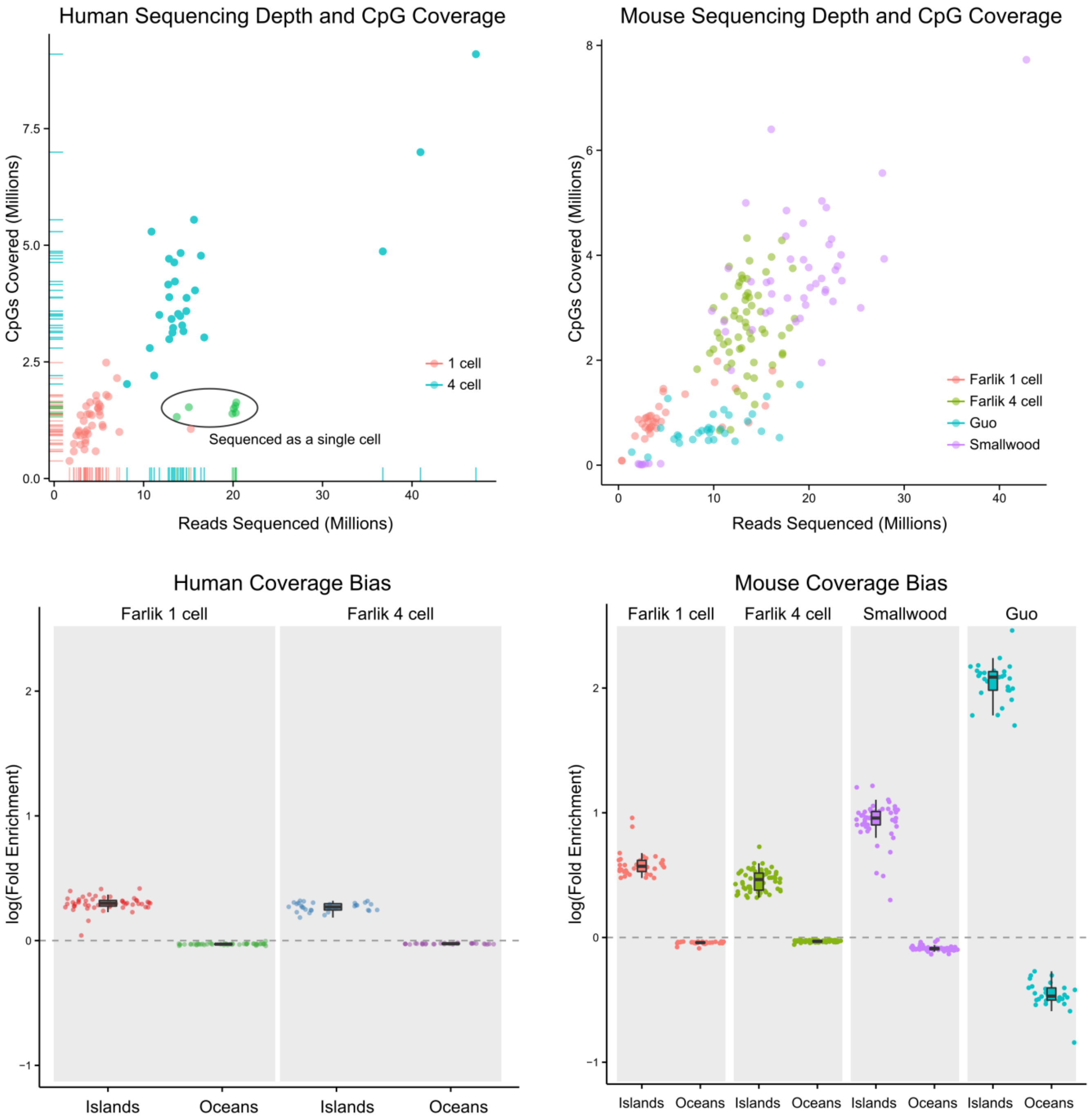

Figure S2. Coverage and biases of single-cell methylome sequencing protocols, Related to Figure 1

The top panels show scatterplots for the relationship between sequencing depth and CpG coverage for human and mouse samples. The bottom panels show the log coverage bias in CpG islands versus CpG-depleted tiling regions ("oceans"). The mouse diagrams include data not only for our scWGBS method, but also for the single-cell RRBS and single-cell PBAT protocols that were published recently (Guo et al., 2013; Smallwood et al., 2014), whereas single-cell methylomes for human cells are currently available only from our study.

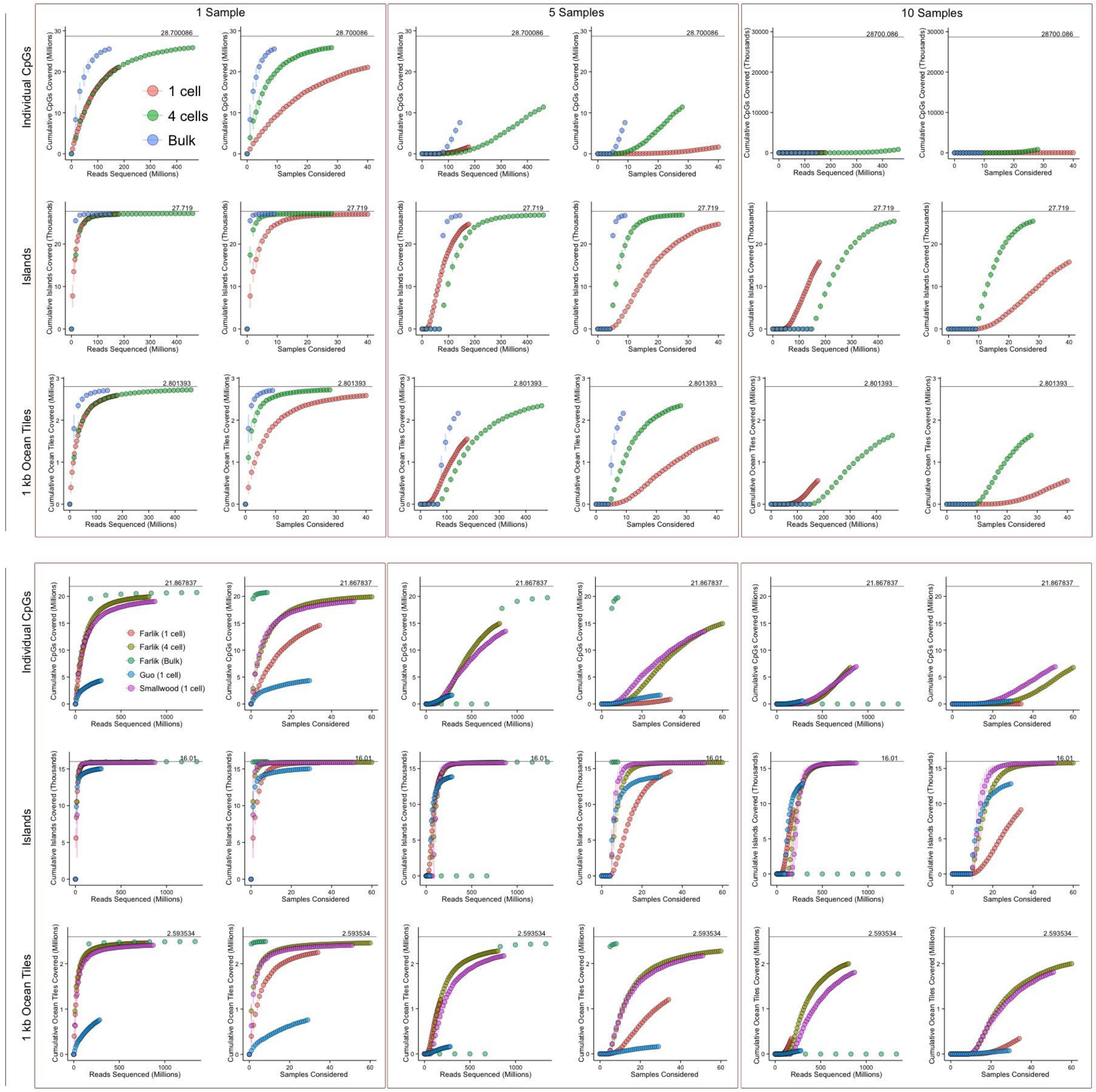

Figure S3. Coverage saturation analysis for human and mouse samples, Related to Figure 2

Saturation plots for human 1-cell, 4-cell, and bulk samples. These plots show the cumulative number of covered individual CpGs (top row), CpG islands (middle row), and 1-kilobase tiling regions located outside of CpG islands (“oceans”, bottom row) when combining many individual samples. The diagrams in the two leftmost columns count an entity (i.e., a CpG dinucleotide, CpG island, or 1-kiloase tiling region) if it is covered in at least 1 individual sample; the diagrams in the center columns require at least 5 samples; and the diagrams in the two rightmost columns require at least 10 samples to constitute coverage. Saturation plots for human (upper panels) and mouse (lower panels) 1-cell, 4-cell, and bulk samples are shown, and the mouse plots also include data for the single-cell RRBS and single-cell PBAT protocols that were published recently (Guo et al., 2013; Smallwood et al., 2014).

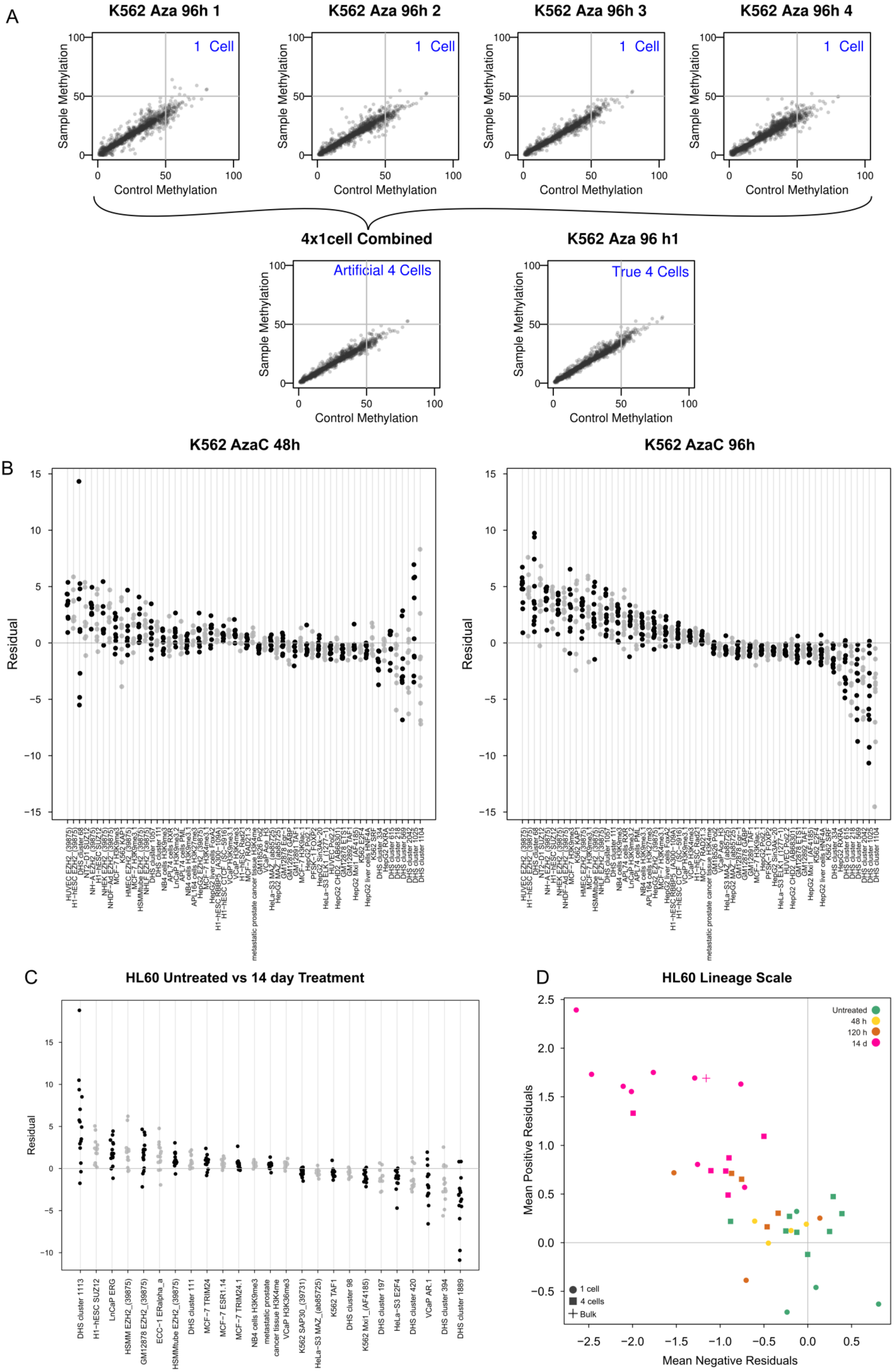

A
K562 Aza 96h 1
K562 Aza 96h 2
K562 Aza 96h 3
K562 Aza 96h 4
1 Cell
Sample Methylation
Control Methylation
4x1cell Combined
Artificial 4 Cells
K562 Aza 96 h1
True 4 Cells
B
K562 AzaC 48h
K562 AzaC 96h
Residual
C
HL60 Untreated vs 14 day Treatment
Residual
DHS cluster 1113
H1-hESC SUZ12
LnCaP ERG
HSMM EZH2_(39875)
GM12878 EZH2_(39875)
ECC-1 ERalpha_a
HSMMtube EZH2_(39875)
DHS cluster 111
MCF-7 TRIM24
MCF-7 ESR1.14
MCF-7 TRIM24.1
NB4 cells H3K9me3
metastatic prostate cancer tissue H3K4me
VCaP H3K36me3
K562 SAP30_(39731)
HeLa-S3 MAZ_(ab85725)
K562 TAF1
DHS cluster 98
K562 Mxi1_(AF4185)
DHS cluster 197
HeLa-S3 E2F4
DHS cluster 420
VCaP AR.1
DHS cluster 394
DHS cluster 1889
D
HL60 Lineage Scale
Untreated
48 h
120 h
14 d
1 cell
4 cells
Bulk
Mean Positive Residuals
Mean Negative Residuals

Figure S4. Variability & region type analysis of K562 and HL60 cells, Related to Figure 4

(**A**) Scatterplots showing four individual 1-cell samples (top row), analogous to the scatterplots in Figure 4B. Combining the DNA methylation measurements from the four 1-cell samples into a composite 4-cell sample yields a plot with substantially reduced variation (bottom left) that is highly similar to an actual 4-cell experiment (bottom right). (**B**) Plots showing the residuals for genomic region sets in K562 cells treated with azacytidine for 96 hours compared to untreated controls, as in Figure 4F. The lower panel shows the residuals for samples that have been treated for 48 hours only, focusing on the same regions as in the upper panel (defined by the 96 hours analysis). The DNase Hyper-sensitive Site (DHS) clusters are collections of regulatory elements with similar cross-tissue patterns (Sheffield et al., 2013). For a given DHS cluster, the regulatory activity across cell types can be investigated using the following web interface: http://dnase.genome.duke.edu. (**C**) Residual plot identifying region types with characteristic differences in DNA methylation between HL60 cells that were treated with vitamin D3 for 14 days and untreated control samples. (**D**) Lineage scatterplot displaying all samples according to the sum of the significant residuals between HL60 cells treated with vitamin D3 for 14 days and the untreated control samples.

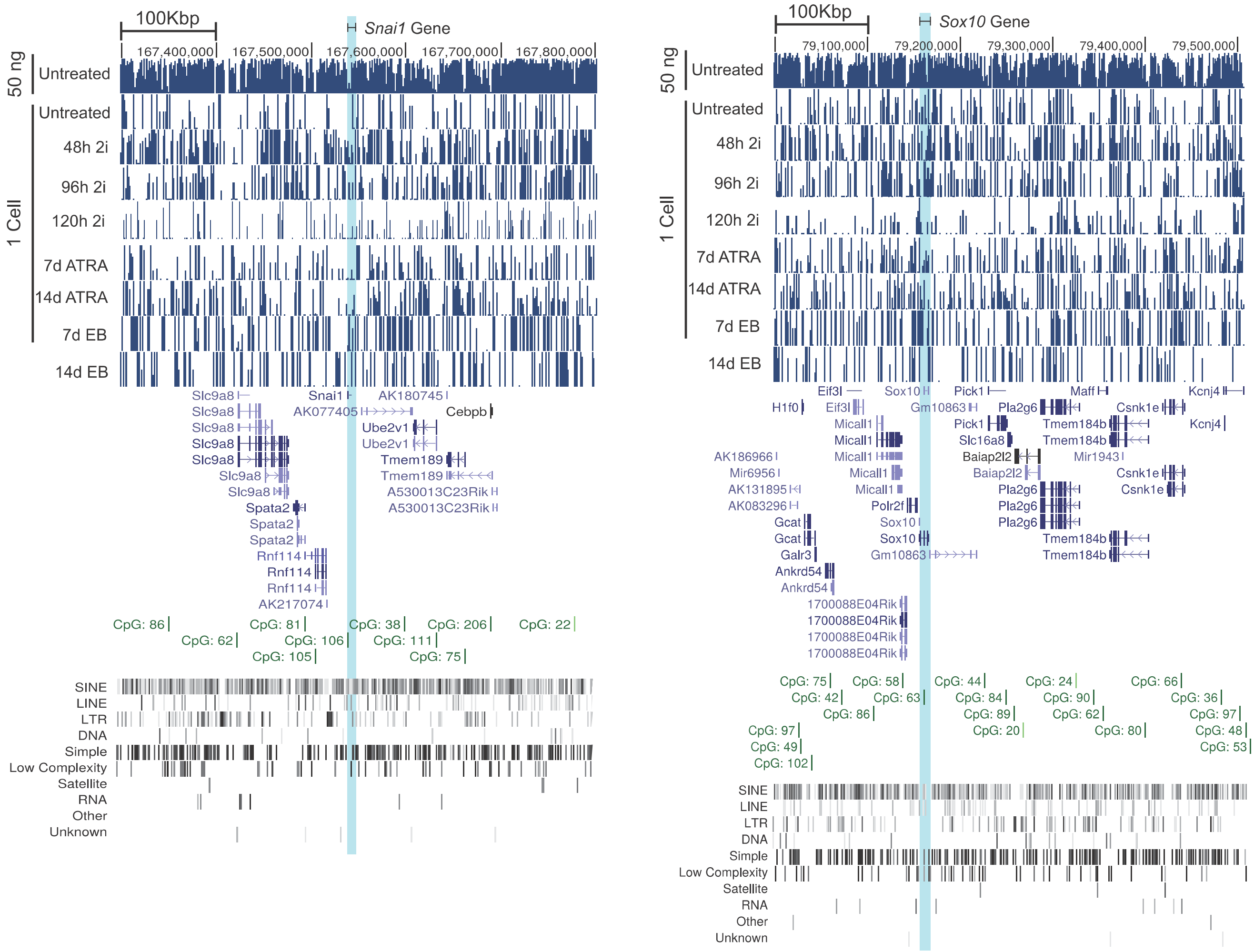

Figure S5: Genome browser view of differential DNA methylation during ES cell differentiation, Related to Figure 5

DNA methylation profile of two 500 kilobase genomic regions surrounding *Snai1* left) and *Sox10* (right).

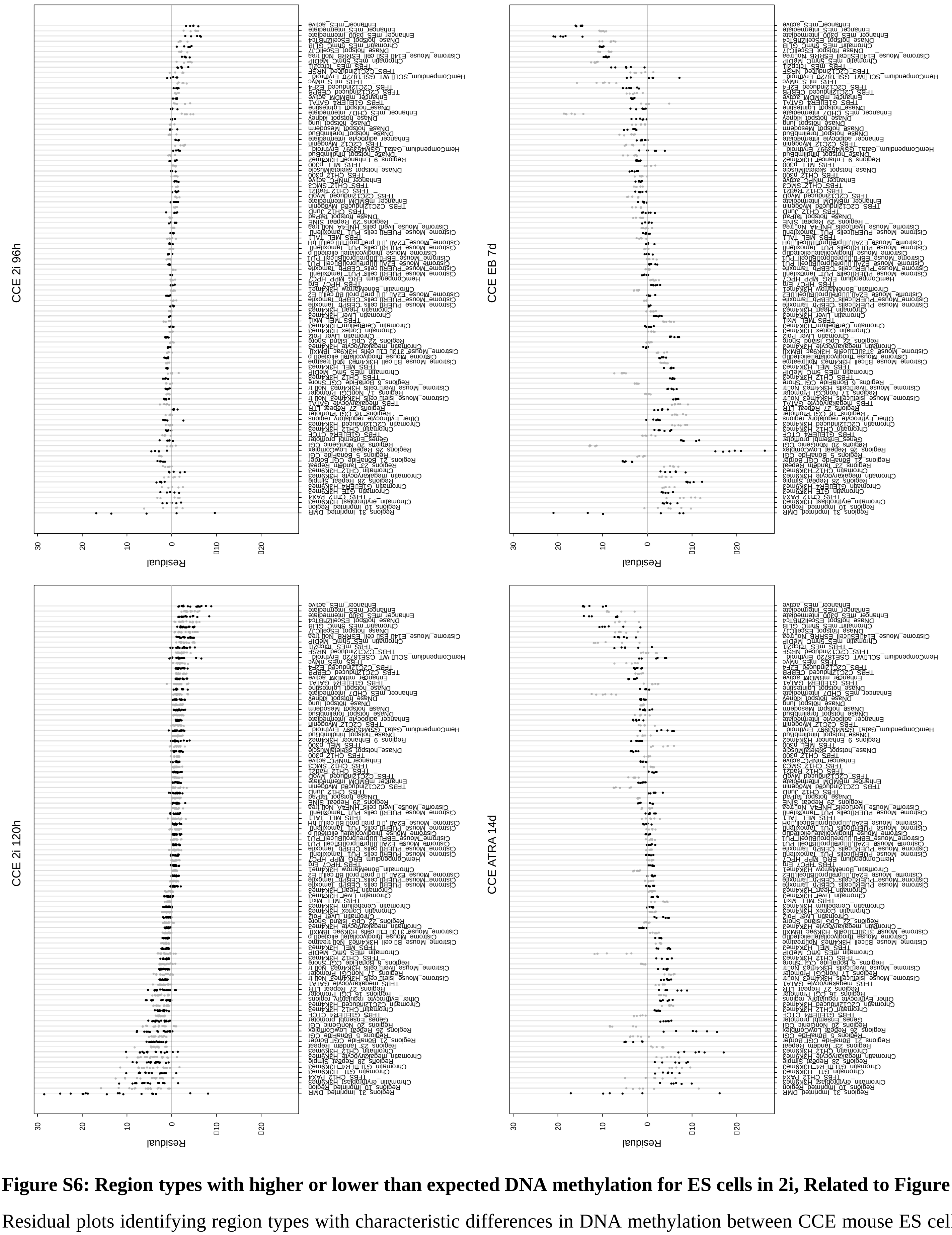

Figure S6: Region types with higher or lower than expected DNA methylation for ES cells in 2i, Related to Figure 6

Residual plots identifying region types with characteristic differences in DNA methylation between CCE mouse ES cells cultured for 120 hours in 2i conditions and untreated control samples (cf. Figure 6A and 6B). The top right panel shows the residuals for samples that have been treated for 96 hours only, focusing on the same regions as in the upper left panel (defined by the 120 hour versus control analysis). The DNase hypersensitive site (DHS) clusters are collections of regulatory elements with similar cross-tissue patterns (Sheffield et al., 2013). For a given DHS cluster, the regulatory activity across cell types can be investigated here: http://dnase.genome.duke.edu.

Table S1. Summary statistics for the methylome sequencing experiments, Related to Figure 1

This table is provided as a separate Excel file

Table S2. Comparison of single-cell methylome sequencing protocols, Related to Figure 2

	**scWGBS** (Bock lab)	**scPBAT** (Kelsey/Reik)	**scRRBS** (Tang lab)
Pre-amplification	No	Yes	No
Maximum total number of PCR cycles	18	19 (5+14)	47 (25+22)
One-tube library preparation	Yes	No	Yes
# of protocol steps[1]	10	16	11
Paired-end sequencing	Yes	Yes[2]	Yes
Compatible with Illumina primers	Yes	No	Yes
Strandedness	Yes	No	Yes
PCR duplicate identification	Yes	No	No
Over-conversion Rate	<2%	N/A	N/A
Under-conversion Rate	<1%	<2%	<2%
Mouse genome coverage:			
Median # of covered CpGs in islands	74,190	450,563	228,171
Median # of covered CpGs in non-islands	761,709	2,921,696	392,009
Median number of reads sequenced	3,558,572	18,620,980	9,572,299
Median bias towards islands	1.8	2.6	8.1
Human Coverage Statistics:			
Median # of covered CpGs in islands	132,046	N/A	N/A
Median # of covered CpGs in non-islands	1,202,639	N/A	N/A
Median number of reads sequenced	4,641,667	N/A	N/A
Median bias towards islands	1.4	N/A	N/A

[1] clean-up was counted as one step

[2] paired-end sequencing was done but alignment treats data as single-end reads to compensate for a high number of hybrid reads
