# Supplementary material for: Single-Cell DNA Methylome Sequencing and Bioinformatic Inference of Epigenomic Cell-State Dynamics
Source: Cell Rep. 2015 Feb 26;10(8):1386–97. doi: 10.1016/j.celrep.2015.02.001 (PMC4542311; doi:10.1016/j.celrep.2015.02.001)
Supplement: Document S2. Article plus Supplemental Information [file mmc3.pdf]

Cell Reports

Resource

Single-Cell DNA Methylome Sequencing and Bioinformatic Inference of Epigenomic Cell-State Dynamics

Graphical Abstract

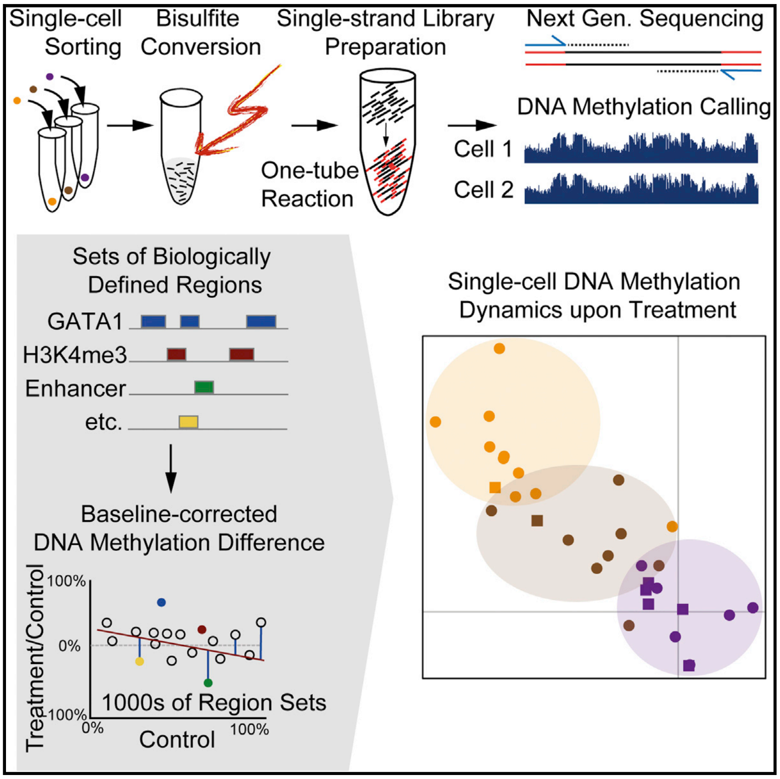

Authors

Matthias Farlik, Nathan C. Sheffield, ..., Johanna Klughammer, Christoph Bock

Correspondence

cbock@cemm.oeaw.ac.at

In Brief

Farlik et al. describe a method for DNA methylation sequencing in very small cell populations (μWGBS) and single cells (scWGBS). Furthermore, they present a bioinformatic method for analyzing low-coverage methylome data and apply this technique to inferring epigenomic cell-state dynamics in pluripotent and differentiating cells.

Highlights

- High-throughput bisulfite sequencing assay for low-input and single-cell samples
- Single-cell methylomes for in vitro models (K562 AzaC, HL60 VitD, and mES 2i/ATRA/EB)
- Bioinformatic method for inferring cell-state dynamics from sparse methylome data
- Identification of genomic region types with consistent changes among single cells

Accession Numbers

GSE65196

Farlik et al., 2015, Cell Reports *10*, 1386–1397
March 3, 2015 ©2015 The Authors
http://dx.doi.org/10.1016/j.celrep.2015.02.001

OPEN ACCESS
CellPress

Cell Reports
Resource

Single-Cell DNA Methylome Sequencing and Bioinformatic Inference of Epigenomic Cell-State Dynamics

Matthias Farlik,[1,4] Nathan C. Sheffield,[1,4] Angelo Nuzzo,[1] Paul Datlinger,[1] Andreas Schönegger,[1] Johanna Klughammer,[1] and Christoph Bock[1,2,3,*]
[1]CeMM Research Center for Molecular Medicine of the Austrian Academy of Sciences, 1090 Vienna, Austria
[2]Department of Laboratory Medicine, Medical University of Vienna, 1090 Vienna, Austria
[3]Max Planck Institute for Informatics, 66123 Saarbrücken, Germany
[4]Co-first author
*Correspondence: cbock@cemm.oeaw.ac.at
http://dx.doi.org/10.1016/j.celrep.2015.02.001
This is an open access article under the CC BY license (http://creativecommons.org/licenses/by/4.0/).

SUMMARY

Methods for single-cell genome and transcriptome sequencing have contributed to our understanding of cellular heterogeneity, whereas methods for single-cell epigenomics are much less established. Here, we describe a whole-genome bisulfite sequencing (WGBS) assay that enables DNA methylation mapping in very small cell populations (μWGBS) and single cells (scWGBS). Our assay is optimized for profiling many samples at low coverage, and we describe a bioinformatic method that analyzes collections of single-cell methylomes to infer cell-state dynamics. Using these technological advances, we studied epigenomic cell-state dynamics in three in vitro models of cellular differentiation and pluripotency, where we observed characteristic patterns of epigenome remodeling and cell-to-cell heterogeneity. The described method enables single-cell analysis of DNA methylation in a broad range of biological systems, including embryonic development, stem cell differentiation, and cancer. It can also be used to establish composite methylomes that account for cell-to-cell heterogeneity in complex tissue samples.

INTRODUCTION

Cellular differentiation is accompanied by widespread epigenome remodeling. Changes in epigenetic marks such as DNA methylation and histone modifications are being studied with genome-wide assays (Bernstein et al., 2007; Rivera and Ren, 2013), which have advanced our understanding of epigenomic cell states. However, current assays typically require thousands to millions of cells per experiment, making it difficult to study rare cell populations and cell-to-cell heterogeneity. Recent advances in single-cell RNA sequencing demonstrate the value of a higher resolution view (Sandberg, 2014) and suggest that methods for single-cell epigenome mapping could promote our understanding of epigenetic regulation in development and disease.

Whole-genome bisulfite sequencing (WGBS) is the current gold standard for DNA methylation mapping (Cokus et al., 2008; Lister et al., 2008), and it provides coverage for more than 90% of the approximately 28.7 million CpGs in the human genome. The standard WGBS protocol requires micrograms of input DNA, but research is continuing to push this number lower. For example, a tagmentation WGBS protocol reduces the DNA requirements to 20 ng, albeit at the cost of reduced genome-wide coverage (Adey and Shendure, 2012; Wang et al., 2013). As a cost-effective alternative to WGBS, reduced representation bisulfite sequencing (RRBS) yields accurate DNA methylation maps covering 1–2 million CpGs from 30 ng of human DNA (Bock et al., 2010; Gu et al., 2010). RRBS has also been applied to populations of about 100 cells from mouse embryos and oocytes (Smallwood et al., 2011; Smith et al., 2012), yielding data for 1–2 million CpGs out of the approximately 21.9 million CpGs in the mouse genome.

Moving to single-cell analysis of DNA methylation is technically challenging because bisulfite treatment causes extensive DNA damage in the form of nicks, fragmentation, and abasic sites. To overcome this issue, Lorthongpanich et al. (2013) avoided bisulfite treatment altogether and combined methylation-specific restriction enzymes with qPCR, which allowed them to measure DNA methylation in single cells at a few dozen candidate CpGs. Guo et al. (2013) demonstrated genome-scale RRBS in single cells with coverage of 0.5–1 million CpGs. And most recently, Smallwood et al. (2014) extended the post-bisulfite adaptor tagging protocol (Miura et al., 2012) with a whole-genome pre-amplification step, yielding coverage of several million CpGs from single mouse cells.

Here, we describe a WGBS protocol optimized for high-throughput profiling of many single cells. We validated this protocol in both mouse and human cells, and produced the first single-cell methylomes of human cells. To effectively analyze and interpret these data, we developed a bioinformatic method that infers epigenomic cell-state dynamics from low-coverage methylome data. We sequenced over 250 samples in three in vitro models of cellular differentiation. Our results provide a

single-cell perspective on epigenomic cell-state dynamics in pluripotent and differentiating cells, and a broadly applicable method for studying DNA methylation both in single cells (scWGBS) and in very small cell populations (μWGBS).

RESULTS

Low-Input and Single-Cell WGBS

In most WGBS protocols, bisulfite treatment is performed after the sequencing adapters have been ligated, which makes the workflow compatible with standard methods for double-stranded adaptor ligation. Unfortunately, these protocols suffer from high DNA loss because any induced DNA damage between the two ligated adapters can interfere with PCR amplification. We therefore focused our optimizations on an existing protocol that uses post-bisulfite adaptor ligation on 50 ng of input DNA, and we found that we could obtain close to optimal methylome data from 6 ng of input DNA (5.8% PCR duplicate read rate, as compared with 1.9% for 50 ng).

To explore the feasibility of sequencing single cells using our optimized protocol, we established a fluorescence-activated cell sorting (FACS)-based workflow that sorts defined numbers and combinations of human and/or mouse cells into single wells of 96-well microtiter plates. The cells can then be lysed, bisulfite treated, and prepared for sequencing (Figure 1A). Importantly, the whole process of library preparation following bisulfite treatment and cleanup is performed in a single tube, which minimizes DNA loss and reduces contamination risk. We validated the accuracy of our workflow in several ways. First, FACS plots confirmed that we could distinguish single cells from rare cell doublets in individual wells of 96-well plates (Figures S1A and S1B). Second, we validated the sensitivity and specificity of the sorting by placing a single mouse embryonic stem cell (ESC) into each well of a 96-well plate. When we counted the number of colonies after 10 days, we observed that 78% of the wells contained exactly one colony and no well contained more than a single ESC colony (Figure S1C). Third, we seeded single mouse hematopoietic 32D cells for clonal expansion and counted the number of cells at days 8, 9, 10, and 11. Extrapolation showed that the observed numbers were consistent with exponential growth starting from a single cell (Figure S1D). Fourth, we performed clonal expansion of human leukemic K562 cells starting from 1, 2, 4, and 8 cells, and we observed 2-fold differences in cell number at day 21, reflecting the 2-fold differences in the number of seeded cells (Figure S1E).

Having validated our ability to accurately sort single cells, we tested the μWGBS protocol for starting amounts of 500, 50, 20, 10, 4, and 1 cell(s), using no more than 18 PCR cycles for library amplification. Data quality was acceptable all the way down to single cells, although there was a visible and progressive deterioration in genomic coverage (Figures 1B and S1F). Importantly, multidimensional scaling (MDS) analysis grouped all samples according to cell type rather than cell number (Figure 1C). Even the one-cell samples clustered with the positive control samples that used 50 ng input DNA, and they were clearly distinguishable from all negative controls (which included libraries prepared from zero cells as well as mouse libraries with reads aligned to the human genome). These observations indicate that our protocol can provide accurate DNA methylation measurements all the way down to single cells.

For additional quality control, in most experiments we sorted one human cell together with four mouse cells (or vice versa) into the same well and performed library preparation on these cell pools. By aligning to both genomes, we could compare the ratio of aligned reads with the ratio of sorted cells and discard samples in which the single cell was lost between sorting and library preparation. Most libraries were well within the expected range (examples are shown in Figure 1D), which provides further validation of the accuracy and robustness of our workflow. Moreover, by comparing cell-type-specific signatures of copy-number variation, we could also confirm that the analyses were not confounded by contaminating DNA (Figure 2A). Finally, to monitor the efficiency of bisulfite conversion, we included methylated and unmethylated oligonucleotides as spike-in controls in most of our libraries, and we observed highly consistent bisulfite conversion rates (Figure 2B).

We prepared and sequenced more than 250 individual samples using the described protocol and workflow. Of these, more than 80% passed all quality-control filtering and were included in our analysis, resulting in 82 single-cell methylomes, 89 four-cell methylomes, and 53 other optimization and quality-control samples (Table S1). We sequenced most samples at low coverage. In humans, a median of 4.6 million reads for the one-cell samples and 13.7 million for the four-cell samples resulted in a median CpG coverage of 1.4 million for one-cell samples and 3.7 million for four cell samples (Figures 2C and S2; Table S2).

Deeper sequencing of a single library consisting of one mouse cell and four human cells increased the CpG coverage by 86% (mouse one-cell) and 62% (human four-cell), but at the cost of high PCR duplicate rates. We observed much more pronounced increases in genome-wide CpG coverage when we combined data from several dozen low-coverage one-cell or four-cell samples into a single composite methylome (Figures 2D, 2E, and S3). This approach profits from the low amplification bias of our protocol (Figure S2), and more than 90% of CpGs in the human and mouse genomes can be covered when a few dozen one-cell or four-cell samples are combined (Figures 2D and 2E). We thus concluded that it is more cost-effective to sequence many one-cell and four-cell samples at low coverage rather than sequencing relatively few of these samples to saturation, and we show below that relatively shallow sequencing can be sufficient for analyzing epigenomic cell-state dynamics (cf. Figures 4, 5, and 6). This conclusion is consistent with recent data from single-cell transcriptomics, where low-coverage sequencing of many samples has become a preferred strategy for reconstructing cellular lineages (Jaitin et al., 2014; Pollen et al., 2014).

Analysis of Single-Cell Methylome Dynamics in Human Hematopoietic Cell Lines

Having established a scWGBS workflow that is sufficiently fast, robust, and cost-effective for processing a larger number of samples, we applied our method to two models of epigenome remodeling and cellular differentiation in human hematopoietic cell lines (Figure 3A). MDS analysis of the global

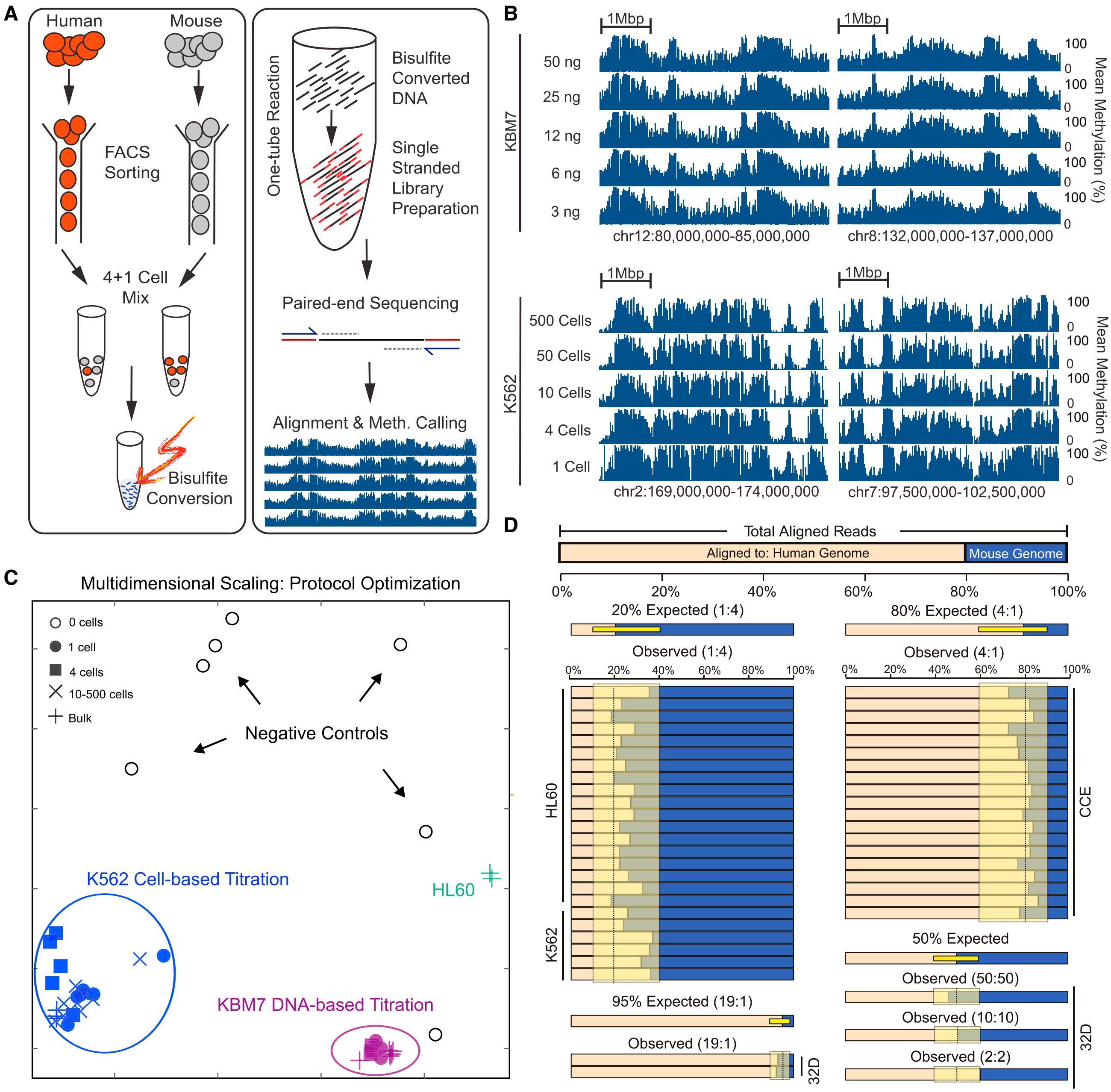

Figure 1. A Workflow for Single-Cell Methylome Sequencing

(A) Overview of the workflow. Defined numbers of human and mouse cells are sorted by FACS and the DNA is bisulfite converted directly on lysed cells, followed by single-strand library preparation and paired-end sequencing.

(B) DNA methylation profiles for four representative genomic regions, plotting the mean DNA methylation levels for windows of 20 kb. The y axis follows the DNA input titration in KBM7 cells (upper panel) and the cell-based titration in K562 cells (lower panel).

(C) Multidimensional scaling (MDS) analysis for average DNA methylation levels of 1-kb tiling regions in DNA-based and cell-based titration samples.

(D) Expected and observed alignment rates for representative samples that passed quality-control filtering. The yellow zones indicate the range of expected values for the ratio of reads aligned to the human and mouse genomes.

See also Figure S1 and Table S1.

similarity among these one-cell, four-cell, and bulk samples showed consistent grouping by cell line (Figure 3B), indicating that the DNA methylation differences between cell lines were stronger than both the variation among single cells (Figure 3B) and the treatment effect in each of the two models (Figures 3C and 3D).

Our first in vitro model was the K562 erythroleukemia-derived cell line treated with azacytidine, an epigenetic drug that is

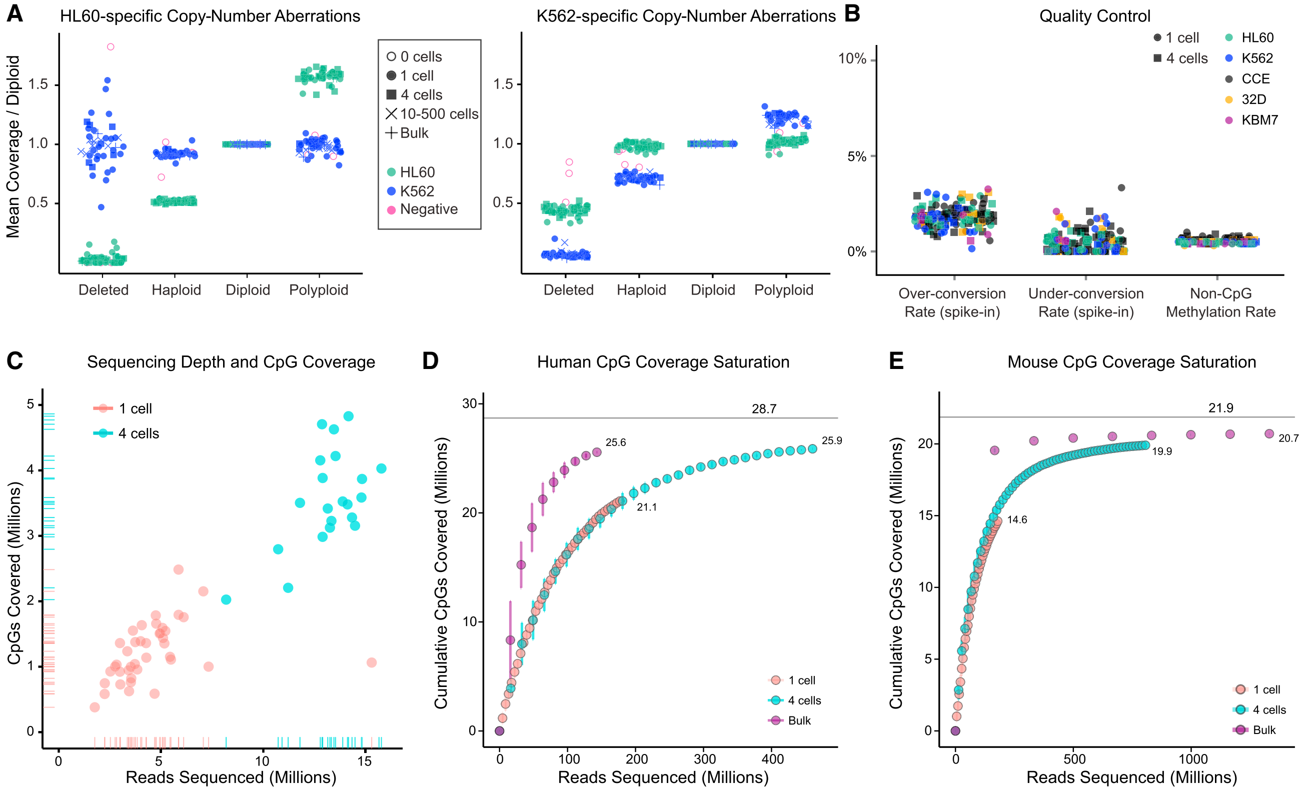

Figure 2. Performance Evaluation of Single-Cell Methylome Sequencing

(A) Strip charts demonstrating concordance between the expected copy-number aberrations in HL60 and K562 cancer cell lines (based on published data) and the observed sequencing coverage.

(B) Estimated rates of bisulfite over-conversion and under-conversion based on methylated and unmethylated oligonucleotides used as spike-in controls.

(C) Scatterplot illustrating the relationship between CpG coverage and sequencing depth for individual one-cell and four-cell human samples. Marginal distributions are plotted as hash marks (rugs) along the axes (see Figure S2 for additional details).

(D) Saturation plot illustrating the relationship between CpG coverage and sequencing depth when combining multiple human samples. Plots show the number of unique CpGs covered (y axis) as a function of aligned reads (x axis). Points are averages across ten iterations adding the individual samples in random order, and the corresponding SDs of CpG coverage are plotted as vertical error bars.

(E) Same as (D) but for mouse samples.

See also Figures S2 and S3 and Table S2.

routinely used in leukemia therapy (Issa and Kantarjian, 2009). Azacytidine depletes DNA methyltransferases, thereby causing widespread reduction in DNA methylation (De Carvalho et al., 2012). We sequenced 31 one-cell and four-cell samples comprising three time points (untreated, 48 hr, and 96 hr) and observed that the samples generally grouped by treatment status (Figure 3C), although there were two treated one-cell samples that clustered with the untreated samples, possibly because these two cells had not divided in the hours after azacytidine treatment. We also observed the expected reduction in global DNA methylation levels in all but these two cells (Figure 3E).

Our second in vitro model was the HL60 cell line, which can be differentiated into monocyte-like cells by treatment with vitamin D3 (Birnie, 1988; Stegmaier et al., 2004). HL60 cells are not known to undergo widespread epigenome remodeling upon vitamin D3 treatment, and the observed morphological changes (Figure 3A) may occur in the absence of major changes in DNA methylation. We analyzed 38 one-cell and four-cell samples that were untreated or treated with vitamin D3 for 48 hr, 120 hr, and 14 days. The one-cell and four-cell samples grouped together according to treatment time (Figure 3D), with distinct directions for the 120-hr and 14-day treatment periods. However, the separation was less clear than in the K562 experiment, and we did not observe any significant changes in global DNA methylation levels (Figure 3E). Overall, the DNA methylation changes that accompanied induced differentiation in HL60 cells were much more subtle than the azacytidine-induced global demethylation of K562 cells.

In both models we also explored whether there were any changes in cell-to-cell variability upon treatment (Figure 3F). To that end, we measured the pairwise Euclidian distances of samples within a treatment group, and we indeed observed an initial increase of cell-to-cell variability upon induction of treatment, followed by a reduction of variability at a later time point. This observation is consistent with the hypothesis that external stimuli such as azacytidine and vitamin D3 initially boost epigenome variability because cells respond individually to treatment,

OPEN
ACCESS
CellPress

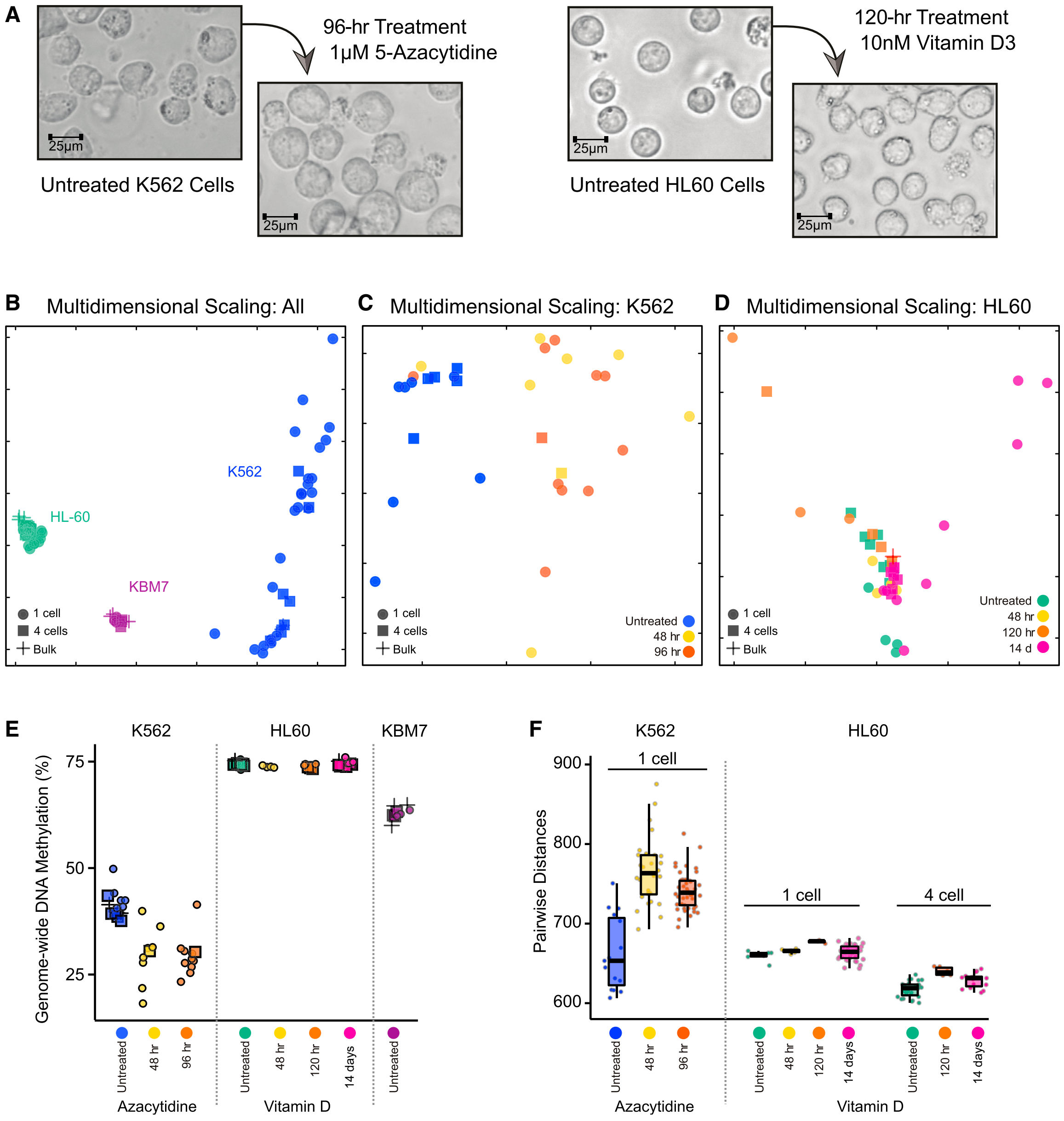

Figure 3. Single-Cell DNA Methylation Dynamics in Drug-Treated and Differentiation-Induced Cell Lines

(A) Bright-field microscopy images of two in vitro models for changes in cell state: K562 cells treated with azacytidine, and HL60 cells treated with vitamin D3.
(B) MDS analysis for average DNA methylation levels of 1-kb tiling regions in human hematopoietic cell line samples.
(C) MDS analysis for K562 cells treated with azacytidine.
(D) MDS analysis for HL60 cells treated with vitamin D3.
(E) Strip charts showing global DNA methylation levels for each sample.
(F) Analysis of pairwise Euclidian distances between individual K562 and HL60 samples.

whereas the variability decreases again when the cells reach their new cell state.

Bioinformatic Method for Inferring Epigenomic Cell-State Dynamics from Single-Cell DNA Methylation Data

Analyzing single-cell methylome data is bioinformatically challenging because the DNA methylation measurements are sparse and discrete. In our initial analysis of global trends in single-cell methylome data (Figure 3), we addressed this issue by averaging DNA methylation levels across 1-kb genomic regions. But more sophisticated methods are needed to obtain insights into the concrete biological processes in such data sets. We thus developed a dedicated bioinformatic method for analyzing single-cell and low-coverage DNA methylation data and inferring epigenomic cell-state dynamics (Figure 4 and Experimental Procedures).

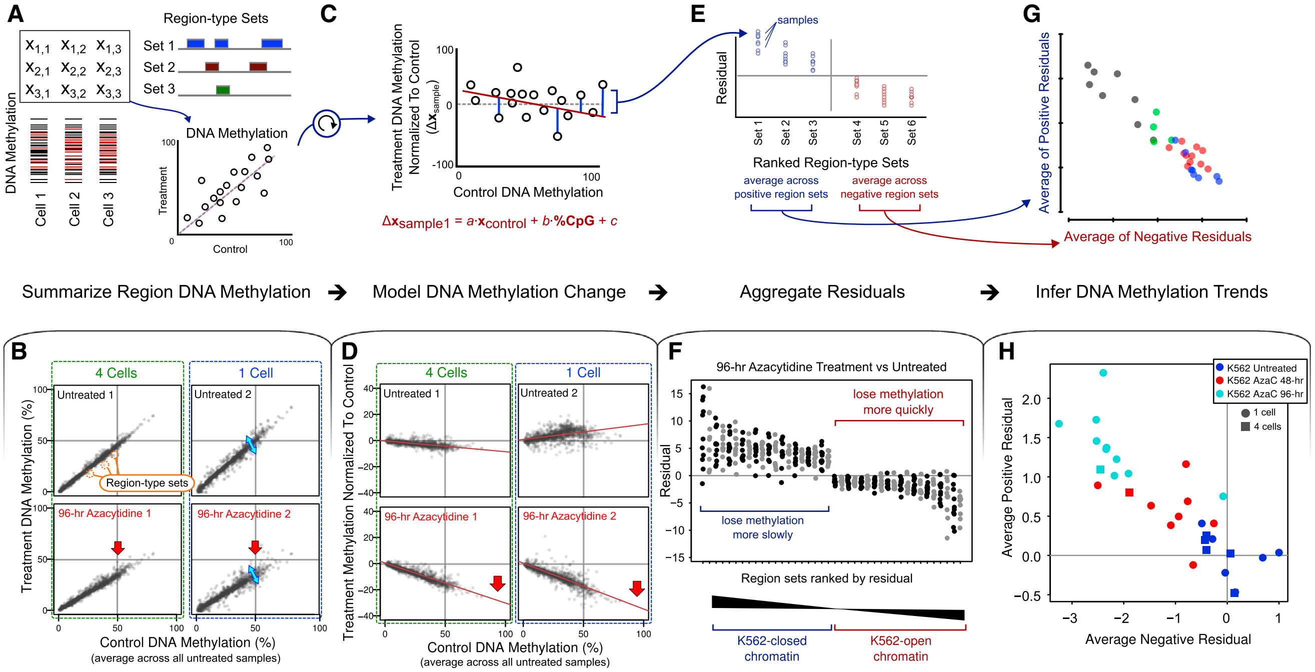

Figure 4. A Bioinformatic Method for Analyzing Low-Coverage and Single-Cell Methylome Data

(A) Aggregation of single-cell DNA methylation data using several thousand biologically defined genomic region sets obtained from public databases.

(B) Scatterplots comparing individual one-cell and four-cell samples against the average calculated across all untreated control samples. The dots represent mean DNA methylation levels across all regions in a given set. Two representative control samples (top row) and two representative 96-hr azacytidine samples (bottom row) are shown.

(C) Correction for systematic global effects among the observed DNA methylation differences. After normalizing to control methylation, a linear model is fitted to model change in DNA methylation level of one individual sample while controlling for the effects of CpG content and DNA methylation level in the untreated control samples.

(D) Scatterplots of the DNA methylation change observed in individual samples plotted against the mean DNA methylation levels among all untreated samples. Each dot represents a region set.

(E) By comparing across individual treated samples, one can identify region sets that show significantly higher (left) or lower (right) DNA methylation levels than expected based on the linear model.

(F) Positive and negative residuals for region sets with significantly higher or lower DNA methylation levels in comparison to the prediction of the linear model in K562 cells. Alternating black and gray coloring is for visualization purposes only. Region sets with consistently positive residuals across samples (left) comprise genomic regions for which DNA methylation is decreasing less quickly with treatment than expected based on the initial DNA methylation state. Negative residuals (right) indicate regions that lose methylation more quickly than expected. The full list of region types is available in Figure S4.

(G) Scatterplot derived by averaging the positive (y axis) and negative (x axis) residuals for each individual sample based on the region sets that are significantly different from expectation.

(H) Scatterplot comparing the 96-hr samples and the untreated controls, with 48-hr samples superimposed.

See also Figure S4.

Briefly, our method measures the degree to which individual one-cell or four-cell samples differ from a set of control samples in terms of average DNA methylation levels across all regions of a given type (such as chromatin binding peaks for p300 or cell-type-specific DNase-hypersensitive sites). Small DNA methylation differences at cell-type-specific transcription factor binding sites have been shown to correlate with cell-type-specific enhancer activity (Bock et al., 2012; Feldmann et al., 2013; Stadler et al., 2011), and combining data across thousands of similar functional elements provides sufficient statistical power to identify small changes in DNA methylation among subsets of such regions. The excellent catalogs of gene-regulatory regions that are now available for the human and mouse genomes provide the basis for extracting biologically relevant signals from our single-cell experiments.

We illustrate our method by analyzing the azacytidine treatment methylomes in more detail (Figure 4). We collected 2,768 experimentally defined region sets from ENCODE and other sources (ENCODE Project Consortium, 2012; Liu et al., 2011; Sheffield et al., 2013), comprising cell-type-specific DNase-hypersensitive sites, regions marked by various histone modifications, transcription factor binding sites, and other types of regulatory regions. For each sample and each region set, we calculated the sample's mean DNA methylation value across all regions of the given region set, resulting in a matrix of samples and region types (Figure 4A). This matrix can be used to visualize the differences of individual samples relative to a defined group of control samples (in this case, all untreated K562 samples). As expected, there were no systematic differences for individual control samples compared with the average of all control

samples, but we observed a clear reduction in global DNA methylation levels for the azacytidine-treated samples (Figure 4B, red arrows). Furthermore, there was higher variability among the one-cell samples compared with the four-cell samples (blue arrows), which disappeared when we combined four one-cell samples into a composite four-cell sample (Figure S4A).

An aggregate analysis of genomic region sets confers sufficient statistical power to identify types of regions that lose DNA methylation faster or slower than average, which can help identify relevant regulatory mechanisms (Figures 4C and 4D). In this analysis, we corrected for two systematic effects that would otherwise dominate the comparison. First, region sets with relatively high DNA methylation levels in the untreated samples were more likely to lose methylation in response to treatment than region sets that were already unmethylated in the untreated samples (as indicated by the trend line and red arrows in Figure 4D). Second, epigenome variability tends to follow different patterns in CpG-rich regions compared with CpG-poor regions (Bock et al., 2008). We corrected for both effects by fitting a linear model for each sample and predicting the expected DNA methylation differences for each region type given the DNA methylation level among the control samples and the CpG content (Figure 4D). By subtracting the expected DNA methylation differences from the observed ones (i.e., by calculating the residuals for the linear model), we identified region types that were more methylated (or less methylated) than one would expect if azacytidine-induced DNA demethylation were entirely unspecific. We then identified region types that were consistently different from the expected value across several individual cells (Figure 4E).

Region types with a consistently positive residual (i.e., those that lose DNA methylation relatively slowly upon azacytidine treatment) were associated with repressive chromatin in K562 cells, whereas regions with consistent negative residuals (i.e., those that lose DNA methylation relatively quickly) comprised lineage-specific enhancer elements and transcription factor binding sites (Figure 4F). This trend was visible not only for the 96-hr samples that were used to define the difference but also for the 48-hr samples (Figure S4B). Finally, we used all region types with statistically significant residuals to visualize single-cell trends (Figure 4G). To that end, we plotted the samples according to their average DNA methylation levels across all region types with significantly positive residuals (y axis in Figure 4H) versus the average DNA methylation levels across all region types with significantly negative residuals (x axis in Figure 4H). The 48-hr samples fell clearly between the untreated samples and the 96-hr samples, supporting our conclusion that azacytidine treatment induces epigenomic changes that are progressive and robustly detectable among single cells (with the exception of the two outliers that were already identified in Figure 3).

We also applied this bioinformatic method to the HL60 samples, which grouped less clearly in the initial MDS analysis (Figure 3D) and did not exhibit global changes in DNA methylation levels (Figure 3E). When we compared untreated samples with cells that had been treated with vitamin D3 for 14 days, we found that a small number of region types differed significantly from the linear model prediction (Figure S4C), allowing us to derive a lineage plot for HL60 differentiation (Figure S4D). Among the region types with elevated DNA methylation levels upon differentiation were ESC enhancer elements, and among those with reduced DNA methylation were DNase-hypersensitive sites specific to blood cell development. These results suggest that differentiation processes without global changes in DNA methylation can exhibit localized patterns of DNA methylation change that are progressive, consistent among single cells, and detectable with our method.

Dissecting Mouse ESC Differentiation and Pluripotency through Single-Cell DNA Methylation Mapping

To test our method in an additional biological system, we applied scWGBS to mouse ESCs cultured in feeder-free serum conditions with leukemia inhibitory factor (LIF) and upon induction of three different stimuli (Figure 5A). First, we induced conversion to naive pluripotency by moving the cells into 2i medium. Second, we induced embryoid body (EB) formation by withdrawing LIF, causing undirected differentiation. Third, we treated ESCs with all-*trans* retinoic acid (ATRA) to induce differentiation in a more directed way.

Among 81 methylomes derived from one-cell and four-cell samples, we observed substantial changes in global DNA methylation levels (Figure 5B). When exposed to 2i medium, most ESCs were significantly demethylated, consistent with previous observations on bulk samples (Habibi et al., 2013). Global DNA methylation levels also decreased after sustained treatment with ATRA (day 14), whereas EB differentiation led to increased DNA methylation levels. MDS analysis of all samples identified one dominant cluster comprising untreated and early-treatment cells, and three treatment-specific clusters branching out in different directions (Figure 5C). These genome-wide trends were also visible at individual loci (Figures 5D and S5). Furthermore, we observed differences between one-cell and four-cell samples only for the 120-hr time point, and these samples were collected on different days in different experiments, suggesting that the speed of DNA demethylation differed between these experiments (Figures 5B and 5C).

For an in-depth analysis, we applied the bioinformatic method outlined in Figure 4 to the mouse ESC methylomes. When we compared untreated samples against all 120-hr 2i samples, we observed consistent changes in DNA methylation for several types of genomic regions (Figure 6A). First, the region set that was most significantly protected from global demethylation consisted of imprinted gene-regulatory regions, indicating that ESCs faithfully maintain genomic imprints in 2i medium despite very low levels of DNA methyltransferase activity. Second, region sets associated with repressive chromatin lost DNA methylation more slowly than expected, whereas enhancer regions of essentially any lineage lost DNA methylation faster. Third, the region sets that lost DNA methylation the fastest overlapped heavily with ESC-specific enhancers, suggesting that their change in DNA methylation levels may be directly driven by the increased activity of the pluripotency regulatory network in 2i medium. These trends were visible not only in the 120-hr samples used to define the difference but also progressively at the 96-hr time point (Figure S6).

When we investigated the DNA methylation dynamics in differentiating cells, we observe strikingly anticorrelated patterns

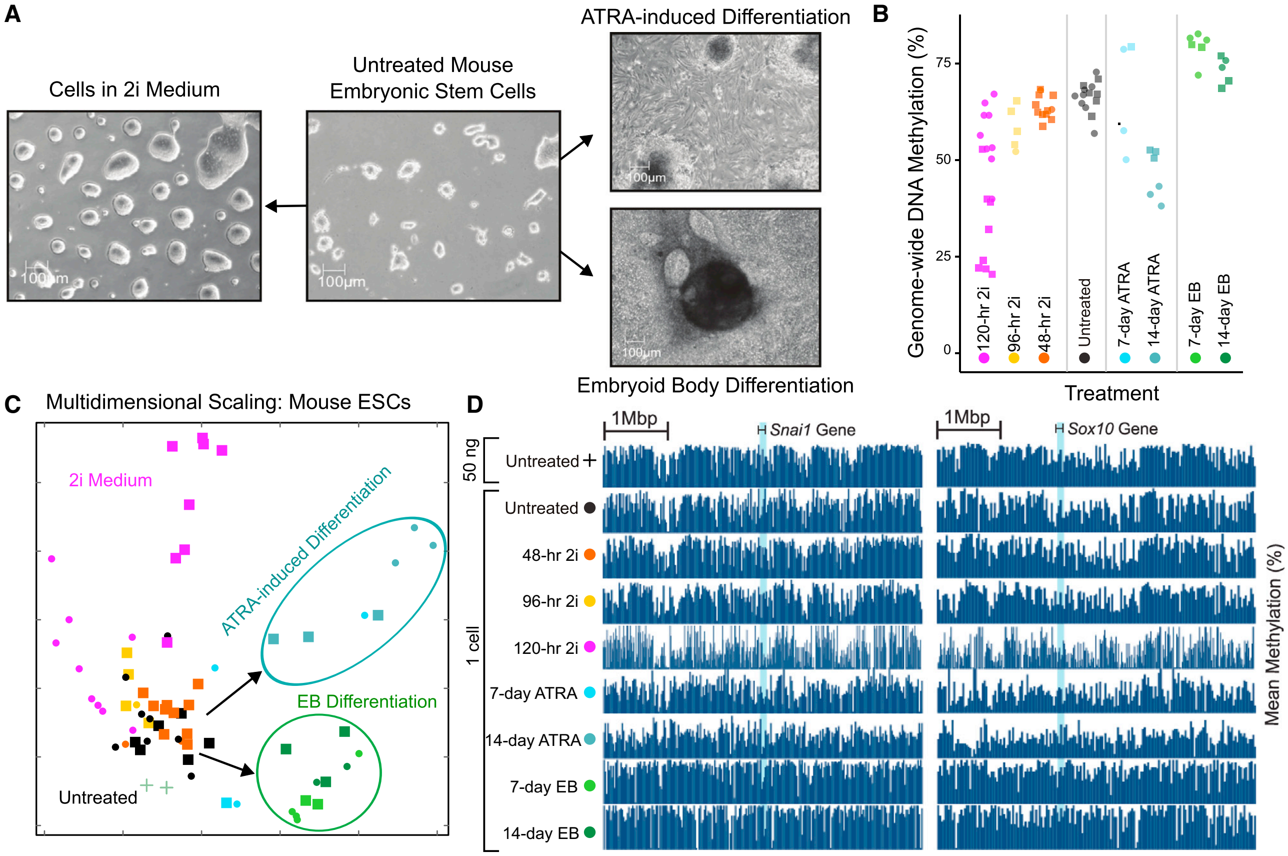

Figure 5. Single-Cell Analysis of Epigenome Remodeling in Pluripotent and Differentiating ESCs

(A) Bright-field microscopy images of mouse ESCs (CCE cell line) cultured in feeder-free serum conditions with LIF (center), after 120 hr in 2i medium (left), and after differentiation induced by ATRA treatment (top right) or by embryoid body (EB) formation (bottom right).

(B) Strip charts showing global DNA methylation levels for each sample and time point.

(C) MDS analysis for average DNA methylation levels of 1-kb tiling regions in mouse ESC derived samples.

(D) DNA methylation profiles for genomic regions associated with two neural differentiation genes, plotting the mean DNA methylation levels for windows of 20 kb. See also Figure S5.

(Figures 6B and S6). Region types that lost DNA methylation most quickly in 2i medium rapidly gained DNA methylation upon EB formation or ATRA treatment, whereas region types that were most protected from DNA methylation loss under 2i conditions had comparatively low DNA methylation levels among the differentiating cells. Only imprinted loci were protected from loss of DNA methylation in either case and in most samples, suggesting that the epigenetic machinery that maintains imprints is able to withstand the changes associated with pluripotency and differentiation in mouse ESCs.

Finally, we plotted all samples according to the average DNA methylation levels among the region types that were identified as significantly different (Figure 6C). The resulting lineage plot organizes all samples along two anticorrelated dimensions of positive residuals (y axis, primarily repressed chromatin) and negative residuals (x axis, primarily open chromatin). It accurately reflects the expected cell states of most of the one-cell and four-cell samples, and is robust enough to compensate for the variable speed of demethylation that we observed among the different 120-hr 2i experiments (cf. Figure 5C). Although this plot was created solely on the basis of differences between 120-hr 2i cells and serum-cultured cells, it logically places the ATRA-induced and EB differentiating cells in the opposite direction. This is not merely a reflection of global changes in DNA methylation, as the ATRA and EB differentiation time courses induce opposite global changes; instead, this observation indicates that our method is able to robustly identify sets of genomic regions from the data that exhibit inverse trends among naive pluripotent and differentiating mouse ESCs.

DISCUSSION

Here we have described an integrated approach for single-cell methylome sequencing and bioinformatic inference of epigenomic cell-state dynamics, and demonstrated its utility in three in vitro models of drug-induced epigenome remodeling, cellular differentiation, and pluripotency. Our workflow includes an optimized protocol for low-input and single-cell bisulfite sequencing that is complementary to two recently described protocols: single-cell RRBS (scRRBS; Guo et al., 2013) and single-cell

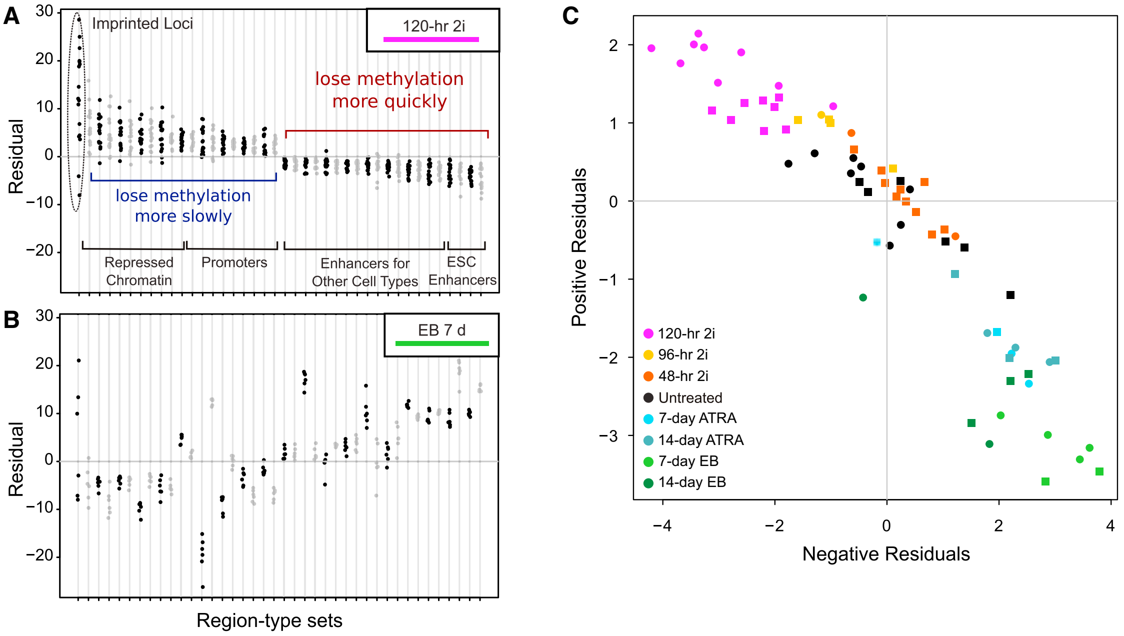

Figure 6. Identification of Key Regulatory Regions and Inference of Epigenomic Cell-State Dynamics for Pluripotent and Differentiating ESCs

(A) Residual plot identifying region types with significant differences in DNA methylation between ESCs cultured in 2i medium for 120 hr and untreated samples as controls. The full list is available in Figure S6.

(B) Residual plot based on the same regions as in (A), but showing 7-day EB samples compared with untreated samples.

(C) Lineage plot displaying all mouse ESC-derived samples according to the sum of the significant residuals between ESCs cultured in 2i medium for 120 hr and the untreated control samples.

See also Figure S6.

post-bisulfite adaptor tagging (scPBAT; Smallwood et al., 2014) (Table S2). Compared with the scRRBS protocol's focus on CpG islands (Guo et al., 2013), our protocol is relatively unbiased and provides cumulative coverage for more than 90% of CpGs in the human and mouse genomes when combining data across experiments (Figures 2D, 2E, and S3). And in contrast to the recently published scPBAT protocol (Smallwood et al., 2014), our protocol does not require any pre-amplification, which offers a number of concrete advantages (e.g., lower reagent cost, less hands-on time, reduced amplification bias, no confounding of strandedness, correct assignment of paired-end fragments, and accurate measurement of PCR duplicates) but comes at the cost of somewhat lower library complexity (Figure S3). We conclude that scWGBS is the method of choice for analyzing large numbers of single cells at low sequencing coverage, scRRBS is useful for comparing CpG islands across single cells, and scPBAT is best suited for deeply sequencing single cells with maximum coverage.

We also developed a bioinformatic method for inferring epigenomic cell-state dynamics from sparse methylome data. Because DNA methylation patterns at regulatory elements of the same type tend to respond similarly to changes in epigenomic cell states, our method gains statistical power by combining data on the level of genomic region sets. We tailored the method specifically to single-cell methylome data, but it could also be useful for analyzing low-coverage WGBS data from large patient cohorts. The three presented case studies demonstrate that our method is able to place single cells on biologically interpretable time-course trajectories, even in cases where changes in DNA methylation are locus specific and non-linear, as illustrated by the human HL60 differentiation and mouse ESC differentiation data.

We anticipate that single-cell methylome sequencing will be useful for a broad range of applications, such as epigenome analysis of heterogeneous organs (Maze et al., 2014) and research on epigenetic drug resistance (Bock and Lengauer, 2012). Because DNA methylation is correlated with histone modifications and chromatin states, it may become possible to use DNA methylation as a surrogate for inferring broader changes in epigenomic cell states. Furthermore, by sequencing and combining dozens of low-input methylomes from the same sample (e.g., one-cell, four-cell, or 20-cell pools), it will be possible to create composite methylomes that provide excellent genome-wide coverage based on relatively few cells and include an inherent assessment of variation. Essentially, composite methylomes redefine the concept of reference epigenome corridors (Bock et al., 2011) in the context of individual samples, thus providing a new type of reference methylome map that can account for tissue heterogeneity and cell-to-cell variation.

EXPERIMENTAL PROCEDURES

Cell Culture and FACS

K562 and HL60 cells were cultured in RPMI medium supplemented with 10% fetal calf serum (FCS) and antibiotics. KBM7 cells were cultured in Iscove's

modified Dulbecco's medium (IMDM) and supplemented with 10% FCS and antibiotics (Bürckstümmer et al., 2013). Mouse ESCs of the CCE cell line were cultured in feeder-free growth conditions using DMEM with the addition of 15% FCS, 2-mercaptoethanol, sodium pyruvate, glutamine, and LIF. 32D cells were cultured in RPMI medium supplemented with 10% FCS, 5 ng/ml mIL3 (eBiosciences, 34-8031-82), and antibiotics. Cell growth and viability were monitored using a CASY cell counter (Roche Diagnostics). FACS was performed using the MoFlo Astrios cell sorter (Beckman Coulter). Prior to FACS, the cells were pushed through a 40 μM cell strainer to ensure the separation of possible aggregates into single cells.

Perturbation Experiments

K562 cells were treated with 5-azacytidine (Toronto Research Chemicals, catalog number A796000) in a final concentration of 1 μM to induce demethylation. HL60 cells were treated with 1α,25-dihydroxyvitamin D3 (Sigma-Aldrich, D1530) in a final concentration of 10 nM to induce differentiation (Ostrem et al., 1987). Naive pluripotency of mouse ESCs was induced by culturing the cells in ESGRO-2i medium (Merck Millipore, SF016) with the addition of 1% FCS. Differentiation of mouse ESCs was induced by treating the cells with ATRA (Sigma-Aldrich, R2625) in a final concentration of 0.3 μM every third day along with complete LIF-free medium exchange. EB formation from mouse ESCs was induced by hanging-drop culture for 5 days, followed by 9 days on gelatinized dishes.

Whole Genome Bisulfite Sequencing

For the positive control samples (which started from 50 ng extracted DNA) and the DNA input titration, DNA was extracted using the Wizard SV Genomic DNA Purification System (Promega, A2360). Bisulfite conversion was performed using the EZ DNA Methylation-Direct Kit (Zymo Research, D5020) according to the manufacturer's protocol, with the modification of eluting the DNA in only 9 μl of elution buffer. For very small cell numbers and single cells, bisulfite treatment was performed directly on lysed cells (rather than on purified DNA) by placing the cells in digestion buffer and performing proteinase K digestion at 50°C for 20 min. In both cases, custom-designed methylated and unmethylated oligonucleotides were added at a concentration of 0.1% to serve as spike-in controls for monitoring bisulfite conversion efficiency. Libraries for next-generation sequencing were prepared using the EpiGnome Methyl-Seq kit (Epicenter, EGMK81312) with the following critical steps: bisulfite-converted genomic DNA was transcribed using tagged random hexamer primers, excess random hexamer primers were digested by the addition of Exonuclease I, terminal tagging was performed to extend the synthesized DNA strand on its 3′ side using elongation blocked and tagged random hexamers, and Illumina-compatible sequencing adapters were introduced through enrichment PCR using primers corresponding to the tagged sequences flanking the random hexamers. For subsequent library amplification, the number of PCR cycles was adjusted according to DNA input or cell number, but never exceeded 18 cycles. The final library was purified twice using Agencourt AMPure XP beads (Beckman Coulter, A63880). Quality control for the final library was performed by measuring the DNA concentration with the QuBit dsDNA HS assay (Life Technologies, Q32851) on QuBit 2.0 Fluorometer (Life Technologies, Q32866) and by determining library fragment sizes with the Experion DNA 1K Analysis kit (Bio-Rad, 700-7107) on the Experion Automated Electrophoresis Station (Bio-Rad, 701-7000). Sequencing was performed on Illumina HiSeq 2000 and 2500 machines.

Data Processing

To prepare the bisulfite sequencing reads for downstream analysis, we developed a bioinformatic pipeline consisting of the following main steps: (1) library adaptor trimming, (2) bisulfite-aware alignment to the human and mouse genomes, (3) DNA methylation calling based on the aligned reads, (4) removal of potential PCR duplicates based on identical start and end positions, (5) filtering of contaminating reads, and (6) calculation of quality measures. Alignment was done with the Bismark Bisulfite Mapper (Krueger and Andrews, 2011), which is a three-letter bisulfite aligner that uses an in silico bisulfite-converted reference genome (Bock, 2012). Library adaptor sequences were removed with Trimmomatic (Bolger et al., 2014). Furthermore, any reads containing fewer than three cytosines outside of a CpG context that had been converted to thymines were discarded, which effectively excluded reads derived from unconverted and contaminating DNA fragments. DNA methylation data and summary statistics were extracted from the Bismark methylation caller output. RnBeads (Assenov et al., 2014) and EpiExplorer (Halachev et al., 2012) were used in an initial analysis of the data set, and the in-depth analysis was performed in R (http://www.r-project.org/). All analyses were done relative to the h19/GRCh37 assembly of the human genome and the mm10/GRCm38 assembly of the mouse genome.

Confirming Cell Identity

Due to the increased risk of contamination when working with very few cells, we sought to confirm the cell line identity of the human one-cell and four-cell experiments. To that end, we retrieved copy-number data for HL60 cells from the Sanger Cell Lines Project (http://cancer.sanger.ac.uk/cell_lines/sample/overview?id=905938). From this data set we extracted hg19 region sets and classified them as deleted, haploid, diploid, or polyploid in HL60 cells. We then filtered any regions that overlapped with potentially ambiguous regions, which we assembled from curated sets of repeats, microsatellites, segmental duplications, and self-chain alignments. We repeated this process for the K562 cell line, using copy-number data from the ENCODE project (http://hgdownload.cse.ucsc.edu/goldenPath/hg18/encodeDCC/wgEncodeHudsonalphaCnv/wgEncodeHudsonalphaCnvRegionsK562V2.bed.gz). Using the copy-number annotated region sets for HL60 and K562 cells, we calculated the average genomic coverage across all region types in each sample by summing the total bases covered in the regions and then dividing by the total number of nucleotides. We normalized each sample by dividing by the coverage in diploid regions for a given cell type.

Saturation Plots and Performance Comparison

We assessed the cumulative CpG coverage of our method by saturation plots. Starting from a single experiment chosen randomly, we calculated how many CpGs were covered. We then added additional experiments randomly and calculated the cumulative number of unique CpGs covered at each step. We repeated this process ten times and then plotted the mean CpG coverage against the number of aligned reads. The analysis was performed separately for human and mouse and for different cell numbers. We also used the saturation plots to compare our method with the scRRBS and scPBAT protocols that were published recently (Guo et al., 2013; Smallwood et al., 2014). To that end, we downloaded published data from the Sequence Read Archive and the NCBI GEO and included these data in the saturation analyses (Figures S2 and S3). The analyses were performed separately for CpG islands and non-CpG-island tiling regions in order to assess the CpG bias of each protocol.

Aggregating DNA Methylation across Region Sets

Changes in DNA methylation tend to affect regulatory regions of the same type in similar ways (Bock et al., 2012; Feldmann et al., 2013; Stadler et al., 2011), allowing us to combine DNA methylation measurements across similar regions to gain statistical power. We assembled sets of biologically defined regions using public data from several sources. For human, we pooled three databases: the ENCODE set of transcription factor binding sites (ENCODE Project Consortium, 2012), the Cistrome database (Liu et al., 2011), and DNase-hypersensitive regions clustered by tissue specificity (Sheffield and Furey, 2012; Sheffield et al., 2013). For mouse, we used a database of region sets that we previously compiled based on ENCODE, Cistrome, and other public databases (Bock et al., 2012). To quantify DNA methylation in a given set of genomic regions, we first calculated mean DNA methylation levels across CpGs for each region individually and then calculated the mean of these means in the whole region set. We filtered out small and low-coverage region sets to reduce background noise (requiring a minimum of 100 and a mean of 200 CpG measurements across samples in a region set).

Modeling Expected Changes in DNA Methylation

To obtain the control methylation for each region type, we calculated the average of the summary scores across all untreated samples in a given experiment. Based on the aggregated DNA methylation values for each region type,

we analyzed differences among region types across samples in response to treatment. For each sample, we normalized the mean DNA methylation of each region type by subtracting the corresponding control value. When we plotted the normalized DNA methylation values against the control values, we observed the expected random variation for the control samples (Figure 4B, top panels), and we observed a negative linear relationship in treated samples (Figure 4B, red arrows). This linear relationship indicates that regions with high initial DNA methylation are more likely to undergo a larger reduction in DNA methylation than regions that are already less methylated in the controls. We therefore applied linear models to control for the non-informative effect of initial DNA methylation values, and also for the impact of CpG density. In a single sample, we modeled the difference in mean DNA methylation for that sample in comparison to the controls as a linear combination of the control DNA methylation plus the mean percentage of CpGs across all regions in each set. By considering the residuals of this linear model, we captured, for each region set, the change in DNA methylation that is explained neither by the initial DNA methylation value nor by the CpG content of the region set. These values are more biologically informative and interpretable than simple differences or ratios because they are no longer confounded by the effects of initial DNA methylation values and CpG density.

Inferring Trajectories of Change in the Epigenomic Cell State

Using the region-set analysis outlined above, we sought to place individual cells on a trajectory defined by the epigenome response to a given treatment. To that end, we selected informative region sets by an unsupervised data-driven approach as follows: First, we identified region sets that were consistently differentially methylated in the treatment samples versus the controls. For each time-series experiment, we used the endpoint samples as the treatment group (K562: 96-hr azacytidine treatment; HL60: 14-day vitamin D treatment; mouse ESCs: 120-hr 2i treatment) and the untreated samples as the control group. We then compared the distribution of residuals in the treatment group versus the controls using t tests performed separately for each region type. We used a pre-defined p value cutoff (0.01) to select up to 20 of the top differential region sets. Positive regions (those that tend to have higher DNA methylation than expected) have significantly greater residuals in treated than in control samples, whereas negative region types have significantly lower residuals in treated than in control samples. We then assigned one summary score for DNA methylation values above expectation and another summary score for DNA methylation values below expectation, which we calculated by averaging the scores for each region set in each category. We used the resulting two scores per sample as x and y coordinates in the "lineage plot," which allows us to visualize where individual samples fall along the treatment-induced trajectory. Although the construction of the lineage plot is susceptible to some degree of overfitting for the two extreme points that are used to select the region sets, it can be validated by assessing whether or not additional time points or treatments that are not used for model selection are placed in a biologically meaningful way. This was demonstrated, for example, for the 2i-based lineage plot that accurately placed the differentiating ESC samples in the opposite direction of the 2i-induced treatment response.

ACCESSION NUMBERS

The DNA methylome data reported in this paper have been submitted to the NCBI GEO and are available under accession number GSE65196.

SUPPLEMENTAL INFORMATION

Supplemental Information includes six figures and two tables and can be found with this article online at http://dx.doi.org/10.1016/j.celrep.2015.02.001.

AUTHOR CONTRIBUTIONS

M.F. performed the experiments. N.C.S. performed the data analysis with contributions from M.F., A.N., P.D., A.S., J.K., and C.B. The manuscript was written by M.F., N.C.S., and C.B.

ACKNOWLEDGMENTS

We would like to thank Florian Pauler and the Denise Barlow lab at CeMM for advice regarding ESC culture and differentiation; the Core Facility Flow Cytometry at the Medical University of Vienna and Dr. Andreas Spittler for their expertise and assistance with FACS sorting; Johanna Hadler, Thomas Penz, and Michael Schuster of the Biomedical Sequencing Facility at CeMM for assistance with next-generation sequencing; Yassen Assenov, Fabian Müller, and Pavlo Lutsik for their support regarding the RnBeads software; and all members of the C.B. lab for their help and advice. This work was performed in the context of the BLUEPRINT project (European Union's Seventh Framework Programme grant agreement no. 282510) and the ERA-NET projects EpiMark (FWF grant agreement no. I 1575-B19) and CINOCA (FWF grant agreement no. I 1626-B22). N.C.S. was supported by long-term fellowships from the European Molecular Biology Organization (ALTF 1431-2013) and the Human Frontier Science Program (LT000211/2014).

Received: May 19, 2014
Revised: November 3, 2014
Accepted: January 29, 2015
Published: February 26, 2015

REFERENCES

Adey, A., and Shendure, J. (2012). Ultra-low-input, tagmentation-based whole-genome bisulfite sequencing. Genome Res. *22*, 1139–1143.

Assenov, Y., Müller, F., Lutsik, P., Walter, J., Lengauer, T., and Bock, C. (2014). Comprehensive analysis of DNA methylation data with RnBeads. Nat. Methods *11*, 1138–1140.

Bernstein, B.E., Meissner, A., and Lander, E.S. (2007). The mammalian epigenome. Cell *128*, 669–681.

Birnie, G.D. (1988). The HL60 cell line: a model system for studying human myeloid cell differentiation. Br. J. Cancer Suppl. *9*, 41–45.

Bock, C. (2012). Analysing and interpreting DNA methylation data. Nat. Rev. Genet. *13*, 705–719.

Bock, C., and Lengauer, T. (2012). Managing drug resistance in cancer: lessons from HIV therapy. Nat. Rev. Cancer *12*, 494–501.

Bock, C., Walter, J., Paulsen, M., and Lengauer, T. (2008). Inter-individual variation of DNA methylation and its implications for large-scale epigenome mapping. Nucleic Acids Res. *36*, e55.

Bock, C., Tomazou, E.M., Brinkman, A.B., Müller, F., Simmer, F., Gu, H., Jäger, N., Gnirke, A., Stunnenberg, H.G., and Meissner, A. (2010). Quantitative comparison of genome-wide DNA methylation mapping technologies. Nat. Biotechnol. *28*, 1106–1114.

Bock, C., Kiskinis, E., Verstappen, G., Gu, H., Boulting, G., Smith, Z.D., Ziller, M., Croft, G.F., Amoroso, M.W., Oakley, D.H., et al. (2011). Reference maps of human ES and iPS cell variation enable high-throughput characterization of pluripotent cell lines. Cell *144*, 439–452.

Bock, C., Beerman, I., Lien, W.H., Smith, Z.D., Gu, H., Boyle, P., Gnirke, A., Fuchs, E., Rossi, D.J., and Meissner, A. (2012). DNA methylation dynamics during in vivo differentiation of blood and skin stem cells. Mol. Cell *47*, 633–647.

Bolger, A.M., Lohse, M., and Usadel, B. (2014). Trimmomatic: a flexible trimmer for Illumina sequence data. Bioinformatics *30*, 2114–2120.

Bürckstümmer, T., Banning, C., Hainzl, P., Schobesberger, R., Kerzendorfer, C., Pauler, F.M., Chen, D., Them, N., Schischlik, F., Rebsamen, M., et al. (2013). A reversible gene trap collection empowers haploid genetics in human cells. Nat. Methods *10*, 965–971.

Cokus, S.J., Feng, S., Zhang, X., Chen, Z., Merriman, B., Haudenschild, C.D., Pradhan, S., Nelson, S.F., Pellegrini, M., and Jacobsen, S.E. (2008). Shotgun bisulphite sequencing of the Arabidopsis genome reveals DNA methylation patterning. Nature *452*, 215–219.

De Carvalho, D.D., Sharma, S., You, J.S., Su, S.F., Taberlay, P.C., Kelly, T.K., Yang, X., Liang, G., and Jones, P.A. (2012). DNA methylation screening identifies driver epigenetic events of cancer cell survival. Cancer Cell *21*, 655–667.

ENCODE Project Consortium. (2012). An integrated encyclopedia of DNA elements in the human genome. Nature *489*, 57–74.

Feldmann, A., Ivanek, R., Murr, R., Gaidatzis, D., Burger, L., and Schübeler, D. (2013). Transcription factor occupancy can mediate active turnover of DNA methylation at regulatory regions. PLoS Genet. *9*, e1003994.

Gu, H., Bock, C., Mikkelsen, T.S., Jäger, N., Smith, Z.D., Tomazou, E., Gnirke, A., Lander, E.S., and Meissner, A. (2010). Genome-scale DNA methylation mapping of clinical samples at single-nucleotide resolution. Nat. Methods *7*, 133–136.

Guo, H., Zhu, P., Wu, X., Li, X., Wen, L., and Tang, F. (2013). Single-cell methylome landscapes of mouse embryonic stem cells and early embryos analyzed using reduced representation bisulfite sequencing. Genome Res. *23*, 2126–2135.

Habibi, E., Brinkman, A.B., Arand, J., Kroeze, L.I., Kerstens, H.H., Matarese, F., Lepikhov, K., Gut, M., Brun-Heath, I., Hubner, N.C., et al. (2013). Whole-genome bisulfite sequencing of two distinct interconvertible DNA methylomes of mouse embryonic stem cells. Cell Stem Cell *13*, 360–369.

Halachev, K., Bast, H., Albrecht, F., Lengauer, T., and Bock, C. (2012). EpiExplorer: live exploration and global analysis of large epigenomic datasets. Genome Biol. *13*, R96.

Issa, J.P., and Kantarjian, H.M. (2009). Targeting DNA methylation. Clin. Cancer Res. *15*, 3938–3946.

Jaitin, D.A., Kenigsberg, E., Keren-Shaul, H., Elefant, N., Paul, F., Zaretsky, I., Mildner, A., Cohen, N., Jung, S., Tanay, A., and Amit, I. (2014). Massively parallel single-cell RNA-seq for marker-free decomposition of tissues into cell types. Science *343*, 776–779.

Krueger, F., and Andrews, S.R. (2011). Bismark: a flexible aligner and methylation caller for Bisulfite-Seq applications. Bioinformatics *27*, 1571–1572.

Lister, R., O'Malley, R.C., Tonti-Filippini, J., Gregory, B.D., Berry, C.C., Millar, A.H., and Ecker, J.R. (2008). Highly integrated single-base resolution maps of the epigenome in Arabidopsis. Cell *133*, 523–536.

Liu, T., Ortiz, J.A., Taing, L., Meyer, C.A., Lee, B., Zhang, Y., Shin, H., Wong, S.S., Ma, J., Lei, Y., et al. (2011). Cistrome: an integrative platform for transcriptional regulation studies. Genome Biol. *12*, R83.

Lorthongpanich, C., Cheow, L.F., Balu, S., Quake, S.R., Knowles, B.B., Burkholder, W.F., Solter, D., and Messerschmidt, D.M. (2013). Single-cell DNA-methylation analysis reveals epigenetic chimerism in preimplantation embryos. Science *341*, 1110–1112.

Maze, I., Shen, L., Zhang, B., Garcia, B.A., Shao, N., Mitchell, A., Sun, H., Akbarian, S., Allis, C.D., and Nestler, E.J. (2014). Analytical tools and current challenges in the modern era of neuroepigenomics. Nat. Neurosci. *17*, 1476–1490.

Miura, F., Enomoto, Y., Dairiki, R., and Ito, T. (2012). Amplification-free whole-genome bisulfite sequencing by post-bisulfite adaptor tagging. Nucleic Acids Res. *40*, e136.

Ostrem, V.K., Lau, W.F., Lee, S.H., Perlman, K., Prahl, J., Schnoes, H.K., and DeLuca, H.F. (1987). Induction of monocytic differentiation of HL-60 cells by 1,25-dihydroxyvitamin D analogs. J. Biol. Chem. *262*, 14164–14171.

Pollen, A.A., Nowakowski, T.J., Shuga, J., Wang, X., Leyrat, A.A., Lui, J.H., Li, N., Szpankowski, L., Fowler, B., Chen, P., et al. (2014). Low-coverage single-cell mRNA sequencing reveals cellular heterogeneity and activated signaling pathways in developing cerebral cortex. Nat. Biotechnol. *32*, 1053–1058.

Rivera, C.M., and Ren, B. (2013). Mapping human epigenomes. Cell *155*, 39–55.

Sandberg, R. (2014). Entering the era of single-cell transcriptomics in biology and medicine. Nat. Methods *11*, 22–24.

Sheffield, N.C., and Furey, T.S. (2012). Identifying and characterizing regulatory sequences in the human genome with chromatin accessibility assays. Genes (Basel) *3*, 651–670.

Sheffield, N.C., Thurman, R.E., Song, L., Safi, A., Stamatoyannopoulos, J.A., Lenhard, B., Crawford, G.E., and Furey, T.S. (2013). Patterns of regulatory activity across diverse human cell types predict tissue identity, transcription factor binding, and long-range interactions. Genome Res. *23*, 777–788.

Smallwood, S.A., Tomizawa, S., Krueger, F., Ruf, N., Carli, N., Segonds-Pichon, A., Sato, S., Hata, K., Andrews, S.R., and Kelsey, G. (2011). Dynamic CpG island methylation landscape in oocytes and preimplantation embryos. Nat. Genet. *43*, 811–814.

Smallwood, S.A., Lee, H.J., Angermueller, C., Krueger, F., Saadeh, H., Peat, J., Andrews, S.R., Stegle, O., Reik, W., and Kelsey, G. (2014). Single-cell genome-wide bisulfite sequencing for assessing epigenetic heterogeneity. Nat. Methods *11*, 817–820.

Smith, Z.D., Chan, M.M., Mikkelsen, T.S., Gu, H., Gnirke, A., Regev, A., and Meissner, A. (2012). A unique regulatory phase of DNA methylation in the early mammalian embryo. Nature *484*, 339–344.

Stadler, M.B., Murr, R., Burger, L., Ivanek, R., Lienert, F., Schöler, A., van Nimwegen, E., Wirbelauer, C., Oakeley, E.J., Gaidatzis, D., et al. (2011). DNA-binding factors shape the mouse methylome at distal regulatory regions. Nature *480*, 490–495.

Stegmaier, K., Ross, K.N., Colavito, S.A., O'Malley, S., Stockwell, B.R., and Golub, T.R. (2004). Gene expression-based high-throughput screening(GE-HTS) and application to leukemia differentiation. Nat. Genet. *36*, 257–263.

Wang, Q., Gu, L., Adey, A., Radlwimmer, B., Wang, W., Hovestadt, V., Bähr, M., Wolf, S., Shendure, J., Eils, R., et al. (2013). Tagmentation-based whole-genome bisulfite sequencing. Nat. Protoc. *8*, 2022–2032.

Cell Reports
Supplemental Information

Single-Cell DNA Methylome Sequencing and Bioinformatic Inference of Epigenomic Cell-State Dynamics

Matthias Farlik, Nathan C. Sheffield, Angelo Nuzzo, Paul Datlinger, Andreas Schönegger, Johanna Klughammer, and Christoph Bock

Supplemental Figures and Tables

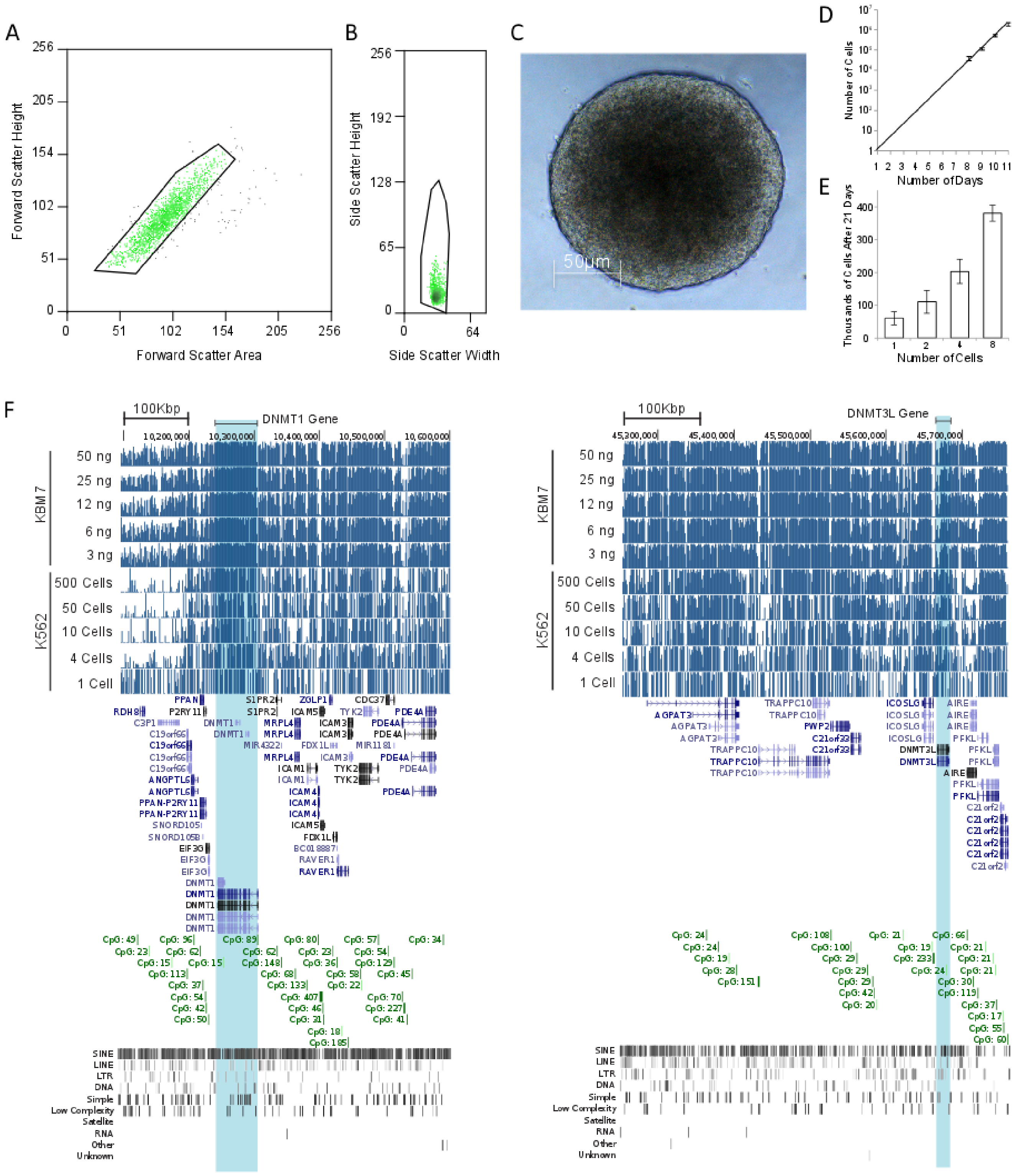

Figure S1. Validation of single-cell sorting, Related to Figure 1

(**A**) Single K562 cells were distinguished from cell doublets (which are visible as a small cloud on the right) based on forward scatter height and forward scatter area. (**B**) Gated single cells were plotted again according to side scatter height and side scatter width (for validation purposes). (**C**) Picture of a representative, single-cell derived ES cell colony. (**D**) The growth of 32D cells was monitored over a period of 11 days. Error bars denote the standard deviation of 10 individual colonies. (**E**) K562 cell growth was monitored for 21 days after initial seeding of 1, 2, 4, or 8 cells. Error bars indicate standard deviation of 10 individual colonies. These results confirm the robustness of the single-cell sorting. (**F**) DNA methylation profiles of two 500-kilobase genomic regions surrounding the DNMT1 gene (left panel) and the DNMT3L gene (right panel).

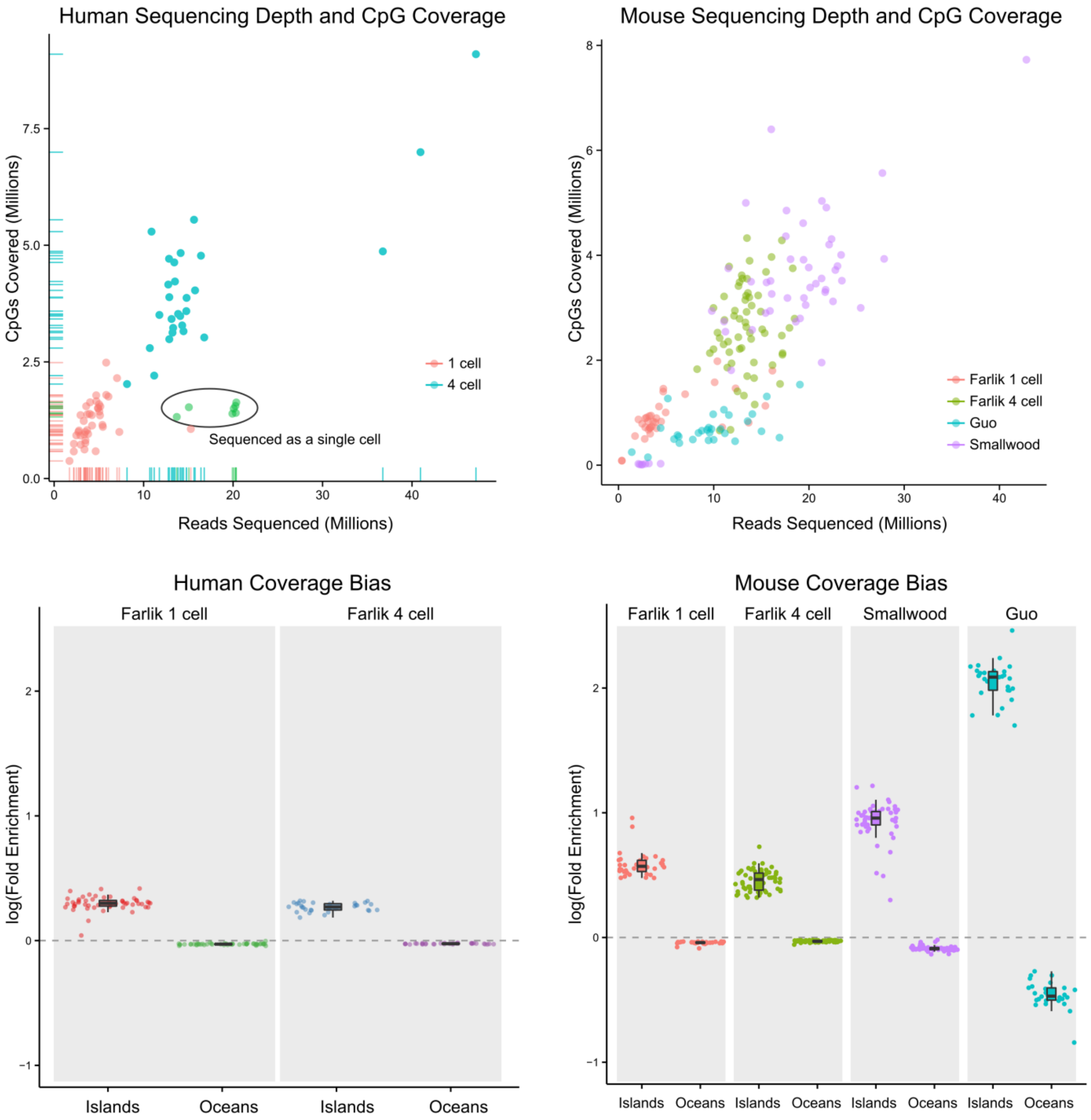

Figure S2. Coverage and biases of single-cell methylome sequencing protocols, Related to Figure 1

The top panels show scatterplots for the relationship between sequencing depth and CpG coverage for human and mouse samples. The bottom panels show the log coverage bias in CpG islands versus CpG-depleted tiling regions ("oceans"). The mouse diagrams include data not only for our scWGBS method, but also for the single-cell RRBS and single-cell PBAT protocols that were published recently (Guo et al., 2013; Smallwood et al., 2014), whereas single-cell methylomes for human cells are currently available only from our study.

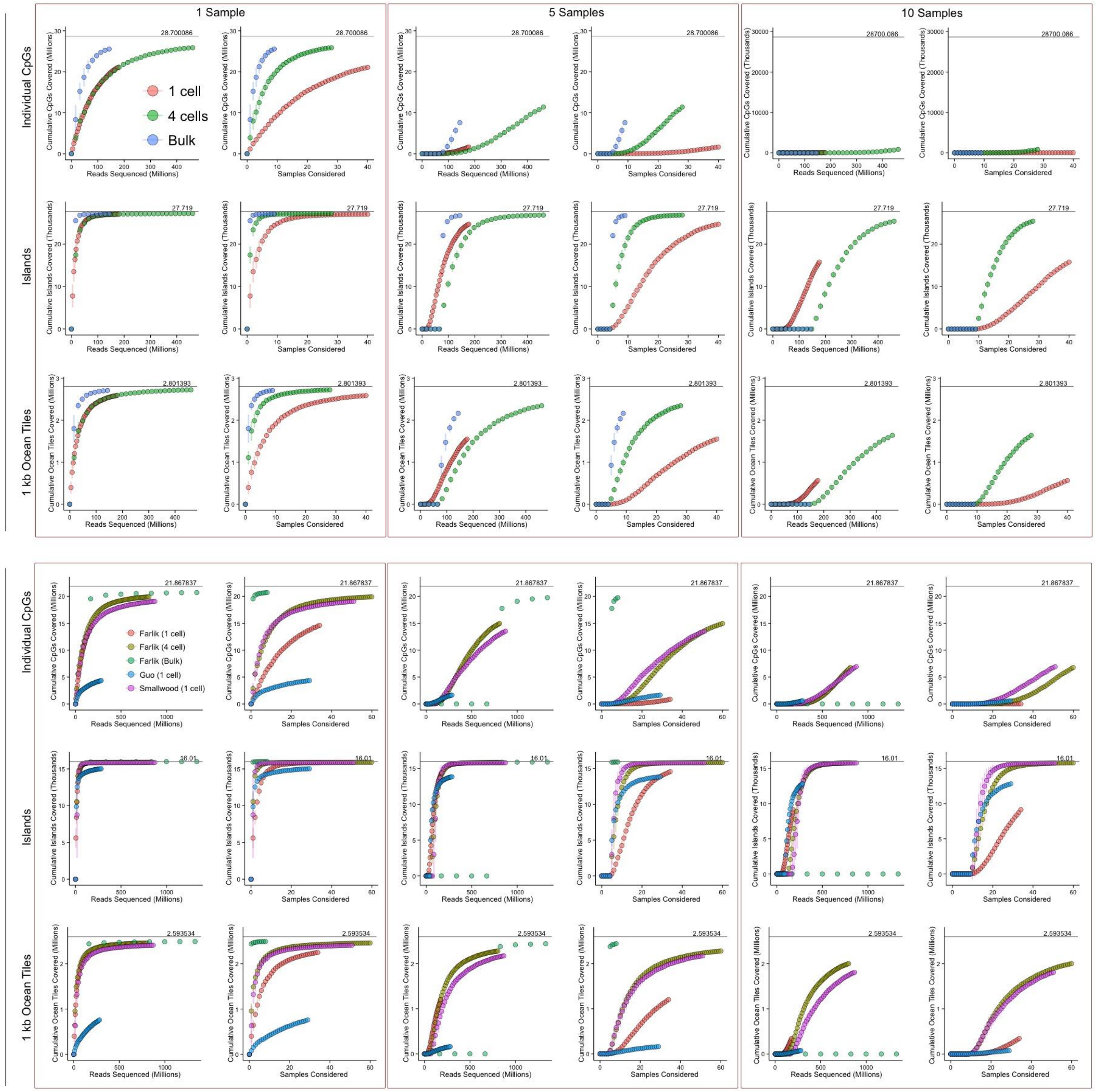

Figure S3. Coverage saturation analysis for human and mouse samples, Related to Figure 2

Saturation plots for human 1-cell, 4-cell, and bulk samples. These plots show the cumulative number of covered individual CpGs (top row), CpG islands (middle row), and 1-kilobase tiling regions located outside of CpG islands (“oceans”, bottom row) when combining many individual samples. The diagrams in the two leftmost columns count an entity (i.e., a CpG dinucleotide, CpG island, or 1-kiloase tiling region) if it is covered in at least 1 individual sample; the diagrams in the center columns require at least 5 samples; and the diagrams in the two rightmost columns require at least 10 samples to constitute coverage. Saturation plots for human (upper panels) and mouse (lower panels) 1-cell, 4-cell, and bulk samples are shown, and the mouse plots also include data for the single-cell RRBS and single-cell PBAT protocols that were published recently (Guo et al., 2013; Smallwood et al., 2014).

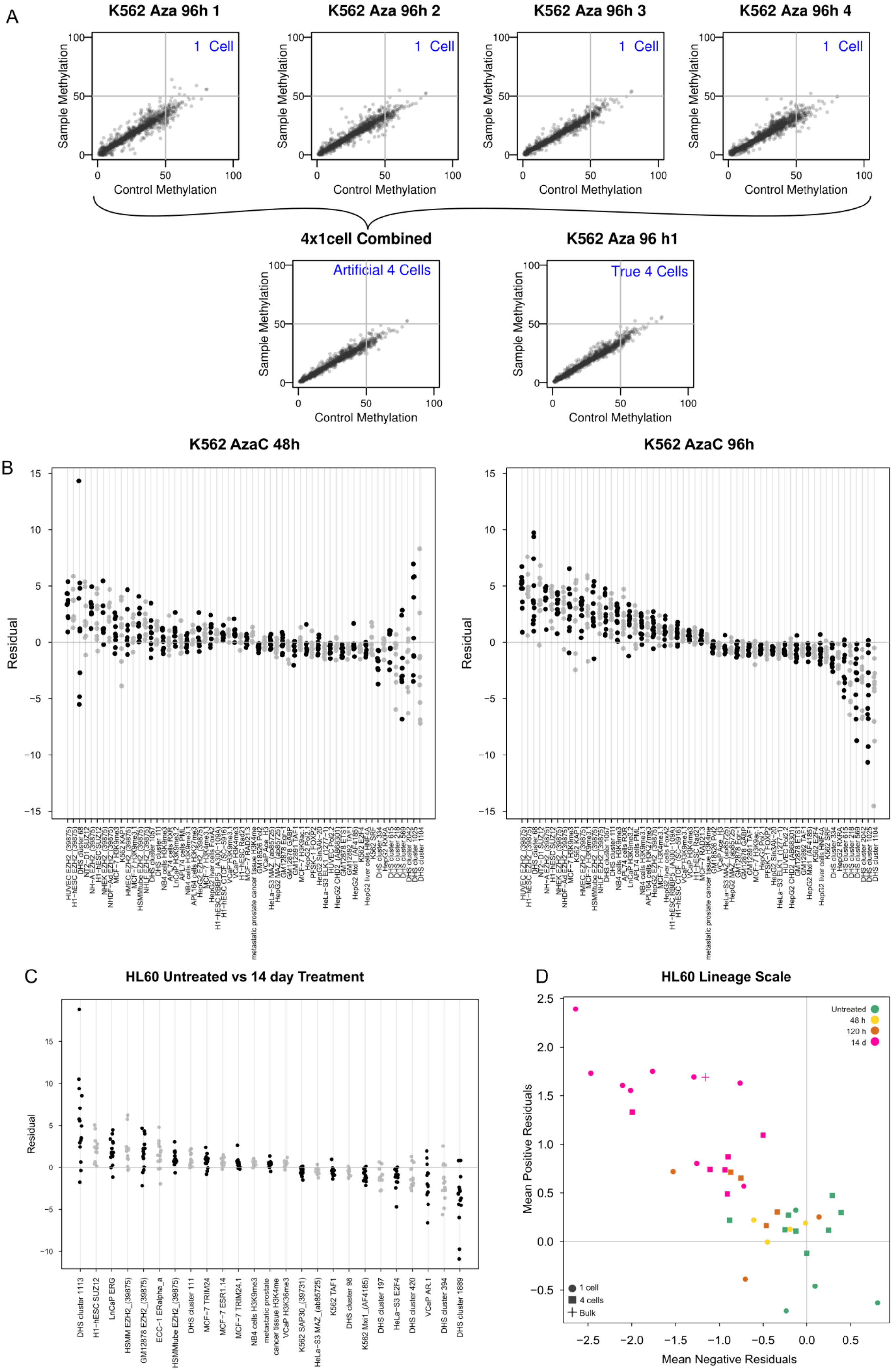

A
K562 Aza 96h 1
K562 Aza 96h 2
K562 Aza 96h 3
K562 Aza 96h 4
1 Cell
Sample Methylation
Control Methylation
4x1cell Combined
K562 Aza 96 h1
Artificial 4 Cells
True 4 Cells
B
K562 AzaC 48h
K562 AzaC 96h
Residual
C
HL60 Untreated vs 14 day Treatment
Residual
DHS cluster 1113
H1-hESC SUZ12
LnCaP ERG
HSMM EZH2_(39875)
GM12878 EZH2_(39875)
ECC-1 ERalpha_a
HSMMtube EZH2_(39875)
DHS cluster 111
MCF-7 TRIM24
MCF-7 ESR1.14
MCF-7 TRIM24.1
NB4 cells H3K9me3
metastatic prostate cancer tissue H3K4me
VCaP H3K36me3
K562 SAP30_(39731)
HeLa-S3 MAZ_(ab85725)
K562 TAF1
DHS cluster 98
K562 Mxi1_(AF4185)
DHS cluster 197
HeLa-S3 E2F4
DHS cluster 420
VCaP AR.1
DHS cluster 394
DHS cluster 1889
D
HL60 Lineage Scale
Untreated
48 h
120 h
14 d
1 cell
4 cells
Bulk
Mean Positive Residuals
Mean Negative Residuals

Figure S4. Variability & region type analysis of K562 and HL60 cells, Related to Figure 4

(**A**) Scatterplots showing four individual 1-cell samples (top row), analogous to the scatterplots in Figure 4B. Combining the DNA methylation measurements from the four 1-cell samples into a composite 4-cell sample yields a plot with substantially reduced variation (bottom left) that is highly similar to an actual 4-cell experiment (bottom right). (**B**) Plots showing the residuals for genomic region sets in K562 cells treated with azacytidine for 96 hours compared to untreated controls, as in Figure 4F. The lower panel shows the residuals for samples that have been treated for 48 hours only, focusing on the same regions as in the upper panel (defined by the 96 hours analysis). The DNase Hyper-sensitive Site (DHS) clusters are collections of regulatory elements with similar cross-tissue patterns (Sheffield et al., 2013). For a given DHS cluster, the regulatory activity across cell types can be investigated using the following web interface: http://dnase.genome.duke.edu. (**C**) Residual plot identifying region types with characteristic differences in DNA methylation between HL60 cells that were treated with vitamin D3 for 14 days and untreated control samples. (**D**) Lineage scatterplot displaying all samples according to the sum of the significant residuals between HL60 cells treated with vitamin D3 for 14 days and the untreated control samples.

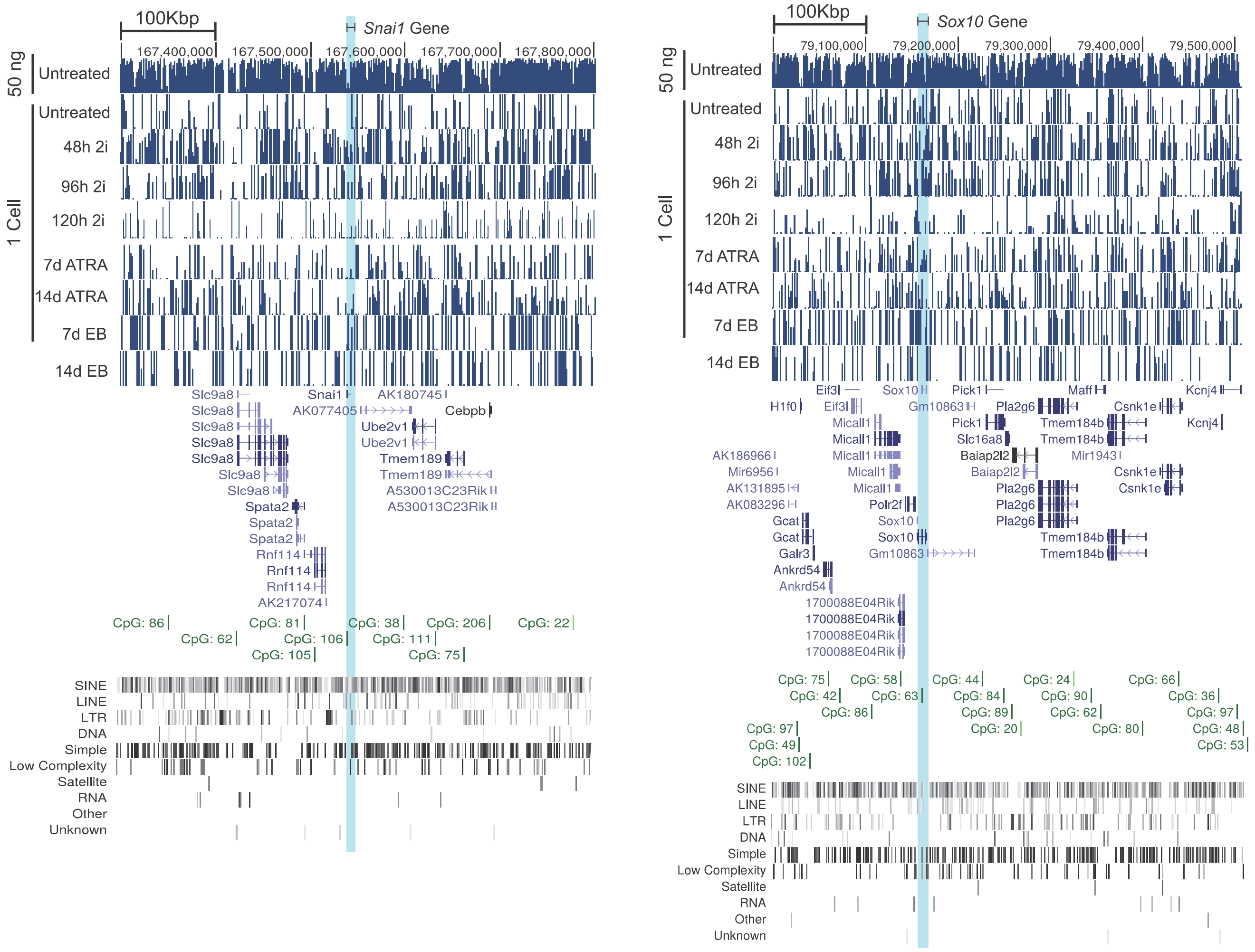

Figure S5: Genome browser view of differential DNA methylation during ES cell differentiation, Related to Figure 5

DNA methylation profile of two 500 kilobase genomic regions surrounding *Snai1* left) and *Sox10* (right).

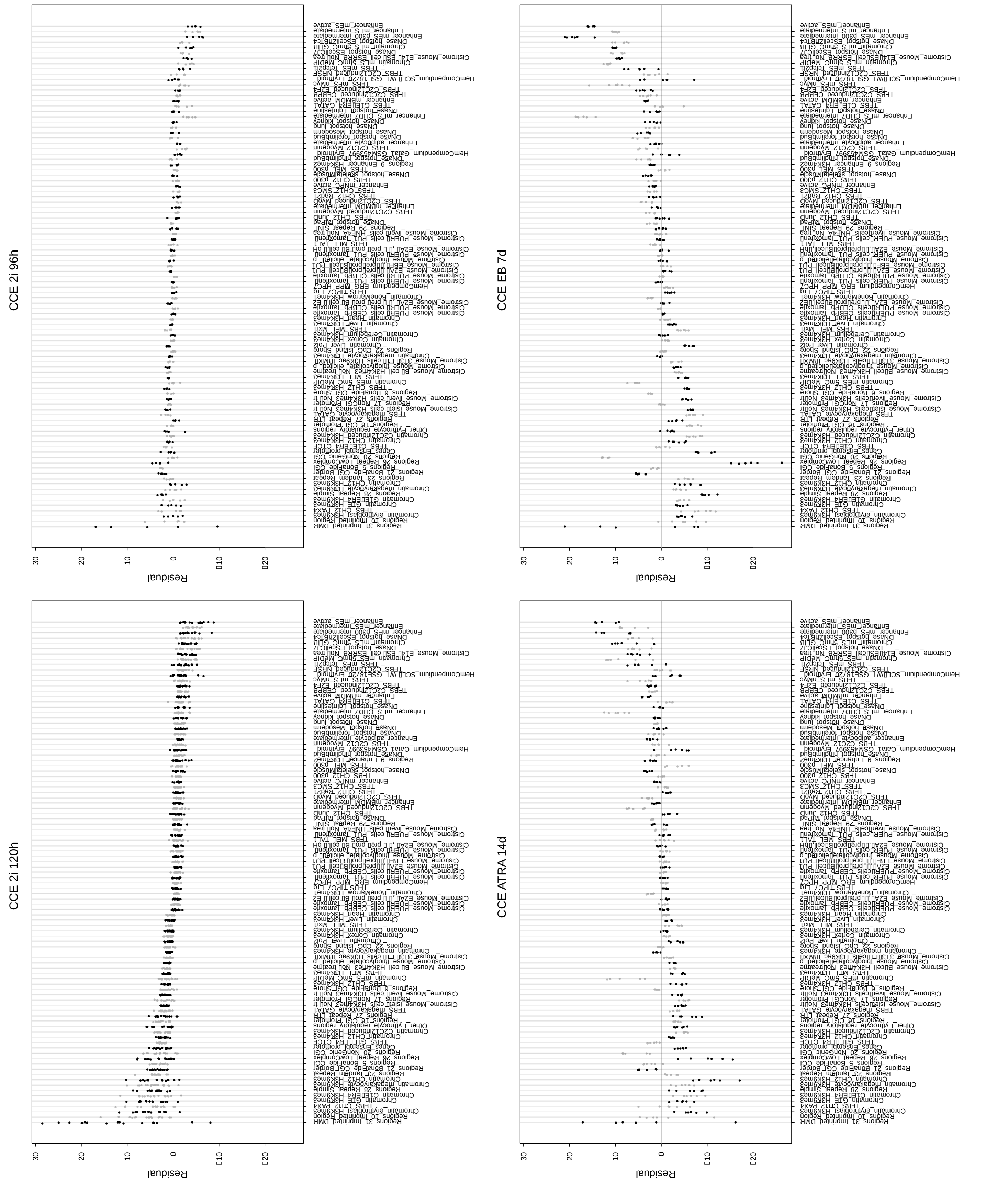

Figure S6: Region types with higher or lower than expected DNA methylation for ES cells in 2i, Related to Figure 6

Residual plots identifying region types with characteristic differences in DNA methylation between CCE mouse ES cells cultured for 120 hours in 2i conditions and untreated control samples (cf. Figure 6A and 6B). The top right panel shows the residuals for samples that have been treated for 96 hours only, focusing on the same regions as in the upper left panel (defined by the 120 hour versus control analysis). The DNase hypersensitive site (DHS) clusters are collections of regulatory elements with similar cross-tissue patterns (Sheffield et al., 2013). For a given DHS cluster, the regulatory activity across cell types can be investigated here: http://dnase.genome.duke.edu.

Table S1. Summary statistics for the methylome sequencing experiments, Related to Figure 1

This table is provided as a separate Excel file

Table S2. Comparison of single-cell methylome sequencing protocols, Related to Figure 2

	**scWGBS** (Bock lab)	**scPBAT** (Kelsey/Reik)	**scRRBS** (Tang lab)
Pre-amplification	No	Yes	No
Maximum total number of PCR cycles	18	19 (5+14)	47 (25+22)
One-tube library preparation	Yes	No	Yes
# of protocol steps[1]	10	16	11
Paired-end sequencing	Yes	Yes[2]	Yes
Compatible with Illumina primers	Yes	No	Yes
Strandedness	Yes	No	Yes
PCR duplicate identification	Yes	No	No
Over-conversion Rate	<2%	N/A	N/A
Under-conversion Rate	<1%	<2%	<2%
Mouse genome coverage:			
Median # of covered CpGs in islands	74,190	450,563	228,171
Median # of covered CpGs in non-islands	761,709	2,921,696	392,009
Median number of reads sequenced	3,558,572	18,620,980	9,572,299
Median bias towards islands	1.8	2.6	8.1
Human Coverage Statistics:			
Median # of covered CpGs in islands	132,046	N/A	N/A
Median # of covered CpGs in non-islands	1,202,639	N/A	N/A
Median number of reads sequenced	4,641,667	N/A	N/A
Median bias towards islands	1.4	N/A	N/A

[1] clean-up was counted as one step

[2] paired-end sequencing was done but alignment treats data as single-end reads to compensate for a high number of hybrid reads
